# Supplementary material for: Customization and acceptability of the WHO labor care guide to improve labor monitoring among health workers in Uganda. An iterative development, mixed method study
Source: PLOS Glob Public Health. 2024 May 13;4(5):e0002780. doi: 10.1371/journal.pgph.0002780 (PMC11090317; doi:10.1371/journal.pgph.0002780)
Supplement: S1 Text — (DOCX) [file pgph.0002780.s001.docx]

**CL001: Adult Clinician, midwife, LCG user**

**I: Thank you so much for accepting to be part of this interview. As I had already told you we wish to know your experience, challenges that you have encountered using the LCG. In all this there in no wrong or right answer but it is just to help us improve it or recommend it for other facilities to use. So I want you to tell me your experience with using this new labour care guide**

R: The new LCG is actually simple compared to the old partograph I do not know how I can elaborate that but all you need is on this paper. You do not need to figure around things. You know with this LCG they give you thing in parts like supportive care …you know the people taking care of the mother in labour, are we giving any pain killers offered in labour, posture…ok, the state the patient is in. Then there is part for the baby to show how the baby is doing and that for vitals of the mother including Temperature, urine output etc, then there is a part for recording contractions, progress of labor and descent. It has a place to record the medications given during the labour process, administered fluids most of which the old partograph didn’t have. There is place for assessment and your plans. Whenever you do anything, you obviously have where to document it. There is also a place for your initials. This time if you do anything you indicate yourself. If you have done something wrong, we will know that it is you. For these the new LCG becomes better than the partograph where you could just plot and go but this one you plot from top to bottom, make your plan and sign your initials. If you have done something wrong, we correct you because we know who. If you have done something good, we also know. At least we can Know the person following up the mother. Comparing with the old partogram, the new LCG is much better.

**I: Comparing the old partograph and this new one: are there any better things on the partograph that are missing on the new tool? Is there anything that you will miss from it or you would wish to maintain??**

R: Really nothing. We left that one behind, completely. The only problem is that the spaces to record are small, especially in the box of assessment and plan, the boxes are small yet people want to write down their plan. You know with labour monitoring; you have to write everything. The small boxes are the only complaints I have, even other midwives have complained of the same.

**I: For you have you experienced that space is small or you are able to write all you need to write**

R: Not really. I don’t think I have too much to write. After all every thing has its space. Here I summarize my plan, simply. Any ways people have kept using the LCG and are getting used to it, this even ceases to be a big problem.

**I: So did they stop complaining? They complained at the beginning…have they now stopped complaining??**

R: Yes, they no longer complain because they are now used. They now learnt how to use the LCG. …*long lough*…. even in training we had case books with small boxes and we had to fill them.

**I: Are there any other challenges?? Apart from the small space for recording, is there any other difficulty you have faced as an individual while using this tool?**

R: As me I am ok. Bu my fellow midwives need like a CME about it and be taught properly. Please talk to the doctor about this. Most have been taught on job. Some still have confusing things with it, but they keep learning. I think staff need a very good training. Like a one-day CME with the doctor because he is a good teacher. One day is enough. For me he taught me in like 30 minutes and I was good to go. I have even taught hers so I am a master now. When I get anything confusing, I still ask the doctor, especially when we are monitoring the mother together. Every patient I monitor there is something new to learn.

**I: So for you from your experience, what are those things that confused you, those that you remember seeking clarification from the doctor**

R: What actually used to confuse be was this second stage plotting. The one that has some weird things, how can I tell you oba… you know I knew that once the mother reaches second stage, you deliver and fill the delivery outcome but in this LCG, you continue monitoring. Some mothers can make it one hour or more in second stage. It doesn’t mean hat when she clocks 10cm the baby just comes. The LCG allows us record labour observations every 15 minutes. I used to be confused. We knew second stage was for recording the outcomes but it is for monitoring

**I: Was there any other challenge?**

R: No.

**I: Back on training, do you think the lack of that sufficient training could have had impact on the midwife’s use of this LCG?**

R: Yes, because some avoid it because they are noy yet confident filling it. You find they have monitored labour, delivered her, mother is fine, baby is fine but they have not filled the LCG

**I: So what do you do for those who refuse to use the LCG**

R: Not that they refuse, but we encourage them to use, and sometimes we are tough on them and record because the records are the only thing that can defend you in court when something bad happens they will chop your academic papers and you become nothing.

**I: Comparing the new tool and the partograph, what is the lay out or design like**

R: The design is ok, one you are used to it becomes better but at the beginning, everything was small. If anything can be done on the size, let it be done. And if you photocopy, they become more invisible. The partograph used to have boxes that were visible and would remain visible even when you photocopied

**I: Or the problem is the photocopier**

R: No. I think when its printed directly from the computer, it is ok. The problem is when photocopied and worse is when you photocopy a photocopy and when midwives have a poor quality LCG, they use it as a reason not to fill the LCG

**I: Is there any other comparison in design??**

R: I will still choose the new LCG

**I: What about if you compare patient identification in the partograph and the new LCG?**

R: the old partograph had similar patient identification details. They are the same

**I: What about the recording of fetal or maternal wellbeing**

R: On the partograph we used to plot with dots in the boxes but the new LCG records the real number, you write what you have counted like 120 0r 140. The boxes clearly show you where to write

**I: What about on progress of labour**

R: I think in the new LCG active labour starts at 5 cm compared 4 cm in the old tool. This section also tells us when the mother delays at a particular point. For example, if the mother is 6 cm and takes more than 5 hours without progressing to 6 cm it means there is a problem. When a woman is 9 cm, it means she is about to deliver and of she stays for example more than 2 hours, then it means there is a problem. On the partograph you would plot until you get to the action line, and be forced to make an action simply because you have reached the action line. This helps you to know when there is a problem, and can easily identify it from the alerts and rectify it before it is too late. In addition, the LCG has prompts for every thing you record, like the box for time shows you space for time in hours, followed by 2 dots indicating the time in minutes. This helps every one monitoring to record standard things.

**I: Which changes or modifications would you recommend to be made on this LCG, is there anything that you think is important, yet missing that you need to be captured?**

R: I think everything is there. Maybe space is not enough but this can be improved by designing it well on A4 sized paper. One other thing, all abbreviations used in filling the LCG are provided at the foot of the page, making the recording of labour observations standard across different uses.

**I: Are all labour abbreviations captured or there are some missing?**

R: They are all there, there is nothing missing. All that we need and use is here. I had forgotten something on contractions. In this LCG when you count 2 contractions you write them as number 2 or 3 compared to the subjective partograph where you shaded to represent the number. Here you write the number and this is clearer. For the duration of contraction, you also record the time the contractions last for example 25 seconds compared with the subjective shading a range of time.

**I: You mentioned that photocopying the LCG is not good, that the letters are small. What exactly did you mean**

R: Yes, letters are small, but what can be done to improve this…but you know capital letters are easier to read than small letters but will they fit in these boxes? If the capital letters fit, then they are better but generally photocopied LCGs are not good

**I: Do you think this LCG captures all the parameters that are needed to adequately needed to monitor labour**

R: Yes, that is a very big yes. It captures everything. Actually, we have more for example urine protein and others were never on the partograph. Like companion, pain relief, fetal heart decelerations, fetal position etc. This is really good

I: Which part of this LCG are more helpful and those that are least helpful

R: Here there is no least or more helpful. There is no less, or upper or more, everything you record here is important, that one I know, you miss out anything you can’t say this is better, each has its role and I can’t say this is better than the other, they work hand in hand. With a bad fetal heart or descent things are not all well.

**I: Tell me about your experience in monitoring mothers in latent labour using the partogram**

R: You don’t monitor a mother using the LCG in latent labour. This mother has not reached 5cm to be started on this tool. You can still examine and monitor but you do not fill the LCG. Filling starts in active labour. As long as I am listening to the baby and the mother is fine, I do not need to start this until we are in active labour

**I: For you what did you think about the changing of active labour from 4 to 5 cm**

R: I think the change is ok. Some of my colleagues who still know that active labour stated at 4 cm still ask where to plot 4cm not knowing it changes to start from 5cm, I think this calls for more training to sensitize midwives that with LCG the definition of active labour changed from 4 to 5 cm. This also disturbed me at first.

**I: Now, you as a midwife, you feel there is no impact of starting to monitor a mother early at 4cm compare to 5cm which is 1 cm later, do you think they are similar?**

R: They are not similar because this is 4 and the other one is 5.

**I: So, don’t you think there are some things that are missed at 4cm when you have not started monitoring**

R: There is nothing missed

**I: How did you feel when you were told that now you are not going to use the old partograph any more, that you were going to start using the new tool. What was that experience**

R: My first question was…why are you making us suffer… *loud prolonged laugh*. To me its is not easy to change from one thing to another, mostly when you are used to the same. When you are used to something, at the beginning you think what you are used to is the best. Its like when you are used to tea with sugar and they say now you will be putting honey in your tea. I had this feeling of… we are used to the partograph, this new one is also for monitoring, then why change

**I: didn’t you resist?**

R: No. I didn’t

**I: Why**

R; I always like new things and they always say in medicine every day is a learning day. Why would I then be stuck on the old thing?? As an in charge, Dr. Mugyenyi called me to office and introduced me to the new tool and asked me to agree to learn this tool together. I saw it and because I trusted him, I started practicing and it worked. When your mind is set to accept a new thing, it can’t be hard.

**I: So you were not happy**

R: Of course not. I needed to understand properly that the tool I learnt from school, from my treasured senior midwives, with very many experiences attached. I had learnt that this is the standard for monitoring a mother in labour but now it will not be any more

**I: If they decided to take you to the old partograph, how would you feel by the way**

R: I will not feel good

**I: Why**

R: Because now I am used to this and its better than the old one. Truth be told, we get inspectors from the city council to check on out practice and when I tell them we are using this new tool, and because they have heard about it yet it’s not in their hospitals, they actually request to be taught how we use it. And when you fill it with them, they agree that its actually simple. Me I get positive things when I meet someone not yet exposed.

I: So did you receive any resources apart from training that you needed to use this tool especially when you were starting to use it. Are there other facilitators

R: No, just the way I told you. Just a call to office, I learnt and I went to teach others

**I: What do you think could have been done better, how should it have been done to make you better prepared to use this tool. Was there a better way**

R: For me this was the best. Teaching me as an individual was good. I was open to ask each and every question I had. In these trainings some times you do not understand and still you do not ask. But me and him figuring out together was great. It worked for me, I learnt and I have been able to teach others

**I: Did you require any incentives or any gifts like money. Does one require to be incentivized to use this tool**

R: No. it would be like you went to school to learn biology and you are asking for the teacher to pay you to learn biology. That would be weird. I knew learning this tool was going to benefit me, really, I do not need a gift to learn. If you start giving me gifts it means you are bribing me.

**I: Ok, what do other people think about your usage of the new tool, like those from the city council**

R: I am telling you they are liking it, only that they haven’t stated using it in their facilities but they are saying its better

**I: How did they know, they looked at it just and said its better??**

R: When they come, we tour them around, they ask me about monitoring labour and I am proud to tell them about the new guidelines and using the new LCG for labour monitoring. They usually tell me they have heard about it but they haven’t used it but wish to learn. One of them asked me for an extra time to brief her about filling the LCG. She filled one, and I filled one. She went away with her copy and promised that when it’s introduced in her facility she will not suffer

**I: What do you think about integrating this new LCG in labour monitoring by the Uganda ministry of health**

R: I think thy should do that as quickly as possible because it’s the best

**I: Is there any other thing that you need to tell me that you could have forgot then??**

R: May be there is need to teach everyone including doctors and obstetricians and gynecologists. They are actually green. Our supervisors sometimes do not know how to use the new tool. They examine mothers and record their findings in the patients file and not on the LCG so they have to do the right things

**I: Is there any other thing you wish to tell me**

R: Just to thank Dr. Mugyenyi for taking this initiative to taking lead in introducing this tool in our practice. It will change our practice

**I: Thank you for your time**

**CL003: Adult Senior Midwife, LCG user and Ward in charge**

**I: Thank you for accepting to talk to us about the LCG. This discussion will not take a lot of your time. We just wish to capture a few details from your experience using the LCG. Tell me your personal experience using the LCG**

R: Me, my experience with the LCG, its better than the partograph because for it, it has many parts including labour supportive care section which doesn’t appear on the partograph. In this one you can easily identify a problem if its there, once you have mastered how to use it. Problems can easily be identified as early as possible. Then again, when you are using it, you know for us being a teaching hospital we have many staffs here and students we have been having challenges of finding someone having delivered a mother, you can’t identify who started the partograph, it is not complete and you do not know the person who has done it. But this LCG there is a space for putting the initials and you can easily the responsible person which is very helpful. And in the other partograph, there was no this part of shared decision making but in this one you discuss with the mother and make a decision and say maybe the contractions are not adequate so we are going to give oxytocin to make hem stronger and ask the mother whether she accepts or declines. When she accepts, you document it as a shared decision and make an appropriate plan. This lacked on the partograph. It has many things which are very good for us and are helping us. In the partograph we used to start monitoring at 4cm even if it was a prime gravida or grand multipara but for this one, we start at 5cm. in the partograph you could examine a mother maybe having a multiparous os and you fit in your 2 fingers and you say 4 cm but at least this one says we start at 5cm, at least you are sure this mother is in active labour. And that part of supportive care it is very important in our labour ward because when you deliver a mother when she has her companion, it becomes easy for her but on a partograph that part is lacking. Here this part continuously reminds you to talk to the mother in labour, asking her whether she has taken tea, where the husband is, know who is around. If the mother-in-law is present, many women here do not culturally allow the mother-in-law to see them giving birth so you do not allow them to enter their delivery room. I think this LCG is better than the partograph.

**I: But in some labour suite, us the husbands are chased away**

R: That’s a mistake to chase away the husband.

**I: But sometimes your wife chases you away that she doesn’t want to see you while delivering**

R: It’s a mistake, but as you have seen, it’s a shared decision. If she says I don’t want to see my husband inside the delivery room, it’s her right

**I: Any challenges you have met while using this LCG?**

R: The only challenge, it needs manpower. When you are like one staff with 5 mothers in labour you cannot manage alone. There are so many things to monitor and record so you may fail if you are handling many mothers at ago. For example, if one goes into second stage and you go to deliver her, what happens to another mother who requires monitoring every 30 minutes. Another challenge is with the size of letters in most of the sections of the LCG. You know as age advances, site problems set in. you find us fidgeting looking for our spectacles to be able to read, yet we are still able to read normal prints. So, the size must be improved. Another good thing with this LCG it has the section of monitoring labour in second stage which was not provided for in the partograph. Another advantage is that you can add another paper if one gets full, as long as the mother and baby are healthy basing on your findings but with the partograph there were those alert and action lines meaning that whenever labour crossed it, it meant that you had obstructed labour and you had to do an action yet the mother may not be having that problem. In the LCG you can continue monitoring as long as everything is normal compared to the partogram which had limits.

**I: So, if I take you back one issue, you said you loke the LCG because of early problem identification. Why is this important**

R: This is important because when you identify a problem early you can intervene. Because whenever there is an alert or a problem, you use a red pen. Even when the midwife monitoring labour changes shift and another one takes shift. When she opens the file and sees red, she knows there is a problem, maybe its breech presentation and will continue to take appropriate action which you wouldn’t have detected easily with the old partograph. This LCG is simpler to use because in the partograph while monitoring contraction you would shade but in this too, you just write the number of contractions in the box corresponding with the time of examination, just that. For the number of contractions, you just write the number fore example if they are 2 just write 2. Only problem is that these processes need time, and because we are handling many mothers at the same time, so its takes time to measure and record everything whenever you have many mothers to monitor.

**I: Apart from time, is there any other problem or challenge you have encountered or faced using this new LCG**

R: The problem of small letters, needs a lot of time and manpower. Other than these there is no problem as an individual. If those are solved and we get big letters I have no problem. By the way, many people are using this LCG without problems, and they are teaching medical and nursing students. They like it.

**I: What do you think, what is that thing about the LCG that makes people get motivated to even teach others including students**

R: You know this is a medical school, if you do not teach the medical student and he or she comes back as an intern, what will you do. There are students who come for experience here and they will be able to monitor labour here, now that here we are using the LCG which is different from their schools, then we have to teach them how to use it.

**I: As for you as an individual, how easy was it to learn to use this tool**

R: For me it wasn’t easy because of my not so good sight [*age related*] but I persisted [*small letters],* being a senior midwife, students and midwives come to you to teach them about the new tool because they want to learn. From the time Dr. Mugyenyi taught us, we have had good experience helping each other, consulting him constantly. We took interest and learnt it. If you asked him that here I do not understand, he takes time and teaches you. From fellow midwives, we took interest to learn because you will be asked, we report about labour monitoring in the meetings. Whenever Dr. Wasswa finds you in labour suite, he asks you, tell me how far is she, where is the filled LCG so we had to learn. Even if there is someone who doesn’t know how to use it, he will come to you as in charge that there is someone in labour suite who doesn’t know how to use the LCG so you have to teach her, so that’s how we all took interest and we have all learnt

**I: Compared to the partogram bout the design. Apart from the things you have mentioned, is there anything you would want to change. Apart from the size of the words, are there any design changes that you recommend**

R: Apart from the small letters, everything is ok.

**I: How do you compare this LCG with the partograph in regards to the patient identification**

R: This is better. It also helps you to identify the problem when it happens, it has a place to record the outcome of labour, it records immunization of the baby, and so it includes everything. It is actually better.

**I: What about when comparing capturing the sociodemographic details. The partograph and the LCG which is better?**

R: This is more detailed.

**I: Details in terms of … [*what*]**

R: You see you are asked to record the names, age, Weeks of amenorrhea, Last monthly period. It captures everything you need to identify the mother.

I: You mentioned that labour monitoring was at 4cm but in this tool it starts at 5cm. how did you feel about that change

R: As I had told you, for us we appreciated the 5cm because sometimes here you get the mothers, here it is a teaching hospital. You get a mother that is 4 cm and after 2 days she is still there, or you are there for 5 hours and she has no contractions, but when the mother if 5c, you are sure she is in labour.

**I: So, for you it’s good to start at 5cm?**

Yes, it is good. Sometimes mothers come claiming to be in labour for long only for you to find she has a multiparous cervix. Whenever you fitted 2 fingers it meant she is 4cm but this could be just a multi parous cervix and not actually labour so she could be advised to return home if she comes from nearby. Then she will return after 1 week later and she delivers well. Don’t you see that that 4 cm thing was not accurate. It was really not correct

**I: Do you think in your knowledge all the parameters or all the requirements for monitoring labour are all captured in this new LCG or there is something, in your experience which you know should have been included but it’s not there**

R: I think everything is captured. Actually, it captures everything we do and all the recommendation up to the first 24 hours, I don’t find anything missing

**I: What would you like changed from this new tool, any modifications?**

R: Just the size of the letters because I know my sight is a problem. I would increase the size of the letters, and increase on the size of the boxes. That is all. Maybe this use of a red pen, not everyone will have a red pen, I even find it difficult to get a red pen for making the duty Rota and whenever I buy one, in a short time I can no longer see it. So, I think circling alert observations with any color available should be sufficient. Once I have circled, it should be sufficient for one to know the observation is an alert and requires action. Not everyone has a red pen.

**I: Supposing the red pens are bought and supplied as part of logistics for implementation of the LCG. Would that help??**

This cannot be sustained on this ward. Even this equipment here on the ward, sometimes you can’t trace them even the large ones like patient monitors can go as far as medical ward, and sometimes Gynaecology ward and you can’t see them for 24 hours. Then what of a pen which can be innocently pocketed

**I: Is there any other challenge**

R: understaffing, but I think this is not directly related to the tool. But it affects its usage, when you have like 10 mothers in labour and you have lie 3 midwives, it becomes difficult. When you increase the number of staff, this will work well. Also, there is need to increase the space, the labour ward is too small for these many mothers. Of recent, pregnant mothers are displaced from their ward by post C-section mothers. We look for mothers in labour and we can’t see them because we do not have beds for them. So, enough staff and space shall improve usage of the LCG

**I: Anything else?**

I find stationary being another big problem, every morning you find the LCGs are finished and when you go to administration, they tell you they do not have papers. This becomes difficult. You start calling Dr. Mugyenyi but he comes and helps whenever he can, to photocopy from the university side. Any you have to have enough copies because when students come and start practicing, it becomes a problem. I think students take them to practice from their hostels. So, you must photocopy enough. So, providing enough stationary will be very helpful. It’s been long when we as a hospital have a challenge with stationery. Patients buy file, books for writing notes etc including discharge forms but for LCG we haven’t required them to photocopy for themselves. Whenever the hospital administration doesn’t help, Dr. Mugyenyi does, or even the office of the head of department whenever they have money. But of recent they do not have. Its Mugyenyi and the hospital administration. So, stationery is a very important.

**I: On the issue of challenges with sight and small letters. You mentioned that you had to learn to use this despite this. You are also in charge and people will come asking you. So, was if from your will or you feel you were forced to learn how to use this tool**

R: No, this was according to my will because I am a midwife, you never know I may go for an interview and they ask me to fill it, anytime you get in the trap just in case you do not know it

**I: Did you receive any resources apart from these printed LCGs. Resources are like the red pens you mentioned. Did you receive any resources?**

R: No. None at all

**I: Have you witnessed anyone refusing to use this LCG? Or has any case of refusal to use LCG been reported to you as an in charge??**

R: No. First of all, it is a tool for monitoring labour, you have to use it. This is a teaching hospital so supervisors always come here and filled LCGs are one of the indicators we report, so people have to make sure that they use it. These people come here and check in patients’ files especially Dr. Wasswa for him he can’t review the mother when he has not seen evidence of monitoring using this LCG. So, it’s a requirement. You have no option.

**I: So, supervision also plays a role**

R: Yes, you know if my supervisor doesn’t find the LCG I am dead do you have to ensure all your patients are monitored and findings documented on the LCG. Even if you do not find it, you have to put it and start filling it

**I: Isn’t that like coercion?**

R: No. As midwives and Obstetricians, we know that is a mandatory requirement

**I: Ok, I understand. As a hospital what are those things that you have in place that make labour monitoring better, like those facilitating conditions**

I: But we do not have them. We do not have them. We do not man power, like now we have one staff on night duty. Post natal mothers, no one is caring, high risk wards no staff is caring. We do not have even a single fetoscope. May be these electronic monitors, they also help us to monitor labour

**I: What facilitated you learning to use this LCG**

R: You know this is a teaching hospital, you have to learn new things. Also, because it’s a requirement to monitor labour, so you have to learn new things. And we loved this tool, people really love it

**I: During that time, you said you had training. How long was it?**

R: I think it took like one and a half hours.

**I: Did you receive like an allowance or refreshments**

R: Not any allowance but for refreshments, yes. In our department it’s a culture to provide refreshments for CMEs and presentations like research proposals. Because people will be sitting for longer than usual so it’s in order to provide a drink. Some have worked night duty with others having come early before breakfast. They can’t sit for an extra hour without a drink

**I: So, he talked about using red pens but he didn’t provide them. What then do you use**

R: We use the usual blue pens to circle all alerts

**I: doesn’t that affect protocol**

R: Of course, but we have nothing to do with that.

**I: What if he ties them somewhere, loke in the bank**

R: [*laugh],* In maternity… people will cut off the string, once someone needs the pen to use, they can do anything

**I: How did others feel when the saw you using the LCG and not the partograph**

R: Here every one is using the LCG. Dr. Mugyenyi empowered everyone. He came to labour ward daily, talked to all of us individually, in groups until when everyone was able to use it. He is the champion for LCG in our hospital, and maybe in the whole Uganda. He gave CMEs and all required trainings

**I: What is your thought on adopting this LCG. This is like it is being piloted, what do you feel about integrating and adopting it in the routine labour monitoring by the Ministry of Health**

My thought is that this tool is very good and it should be adopted, I already told you the good things it possesses. When the people see these good things compared to the partograph they will change like me. For me it should replace the partograph as soon as possible.

**I: Do you see it align to the ministry of health labour monitoring policy**

Yes, it’s very ok, I have no concern

**I: Any other concerns, you could have remembered anything you have left out regarding the discussion and personal experience from using the LCG [as a senior midwife]?**

Number one is to thank Dr. Mugyenyi because he could have done any other thing but due to the love for his work, the delivering mothers. I thank him for bringing this good tool, we see it really helps us and the women in labour in identifying those with specific needs before its too late. Another thing it has helped us to improve documentation because with this tool, if you do not document, you are sure they will catch you. Its not like the partograph where you would scatter some plots of x here and there and you are done. Here you fill in the entire line from beginning up to the last. There is improvement in documentation. Even then when you use it, there is enough space to put most of the things we do, you record the outcome of labour, including immunizations. So the n ext person who comes they will know all that you did on this patient. So its really helpful.

**I: Thank you so much. We are done. We could come back to you if need, thank you for your time.**

**CL004: Adult Clinician, Medical officer and Senior House Officer, LCG trainer**

**I: Thank you for giving us your time to participate in this study. We want just to ask you your experience, personal opinion with this new LCG from the time it was introduced up to now, how do you feel about it**

R: Ok, the LCG from the time it was introduced, at first it was a bit abstract. When we talked about it, my mentor shared with me, the soft copies. It looked like too far away from the reality. It looked so confiscated, so hard to use, you know. and I imagined how it is possible to use it but then as I later on got mentorship, I started getting involved in the webinars and eventually I started training others, then I started using it myself, it looked like when you do not use it you are doing a disservice to your mothers. Initially questions were very many from my colleagues who didn’t have as much as much exposure to the LCG as mine, some of them had fears of saying … when do you act, that the LCG doesn’t have the action line, so when do you act. Then they were saying .. surely were are going to loose more mothers and of course babies but I had to keep coming in and letting them know that actually the reverse is true because with this LCG you easily get the danger signs, we call them alert signs and you were expected to address every single alert as it comes and you are expected not to just look at cervical dilatation and the action lines like it was in the partograph because this mother you are taking care of is not just the cervix and the uterus that is contracting … loud laugh. So, my experience is that this tool if well utilized puts the whole mother picture, it attends to the mother holistically. Its looks at whether this mother has an attendant or labor companion, is this mother taking in oral fluids… you keep telling them to drink tea but are you sure the mother is drinking? Or its just you talking to her. These things were never on the partograph. Caring for the mother is just beyond the cervix that is dilating. So, for me my experience so far is that the LCG is so rich, is intensive and actually looks at the mother in totality.

**I: So, it is holistic**

R: Ya, that’s what I would say

**I: How easy was it for you to learn it**

R: Actually, it was easy. At first my mentor shared it with me and encouraged me to read about it. I looked at them, well, I was more interested in ither things, because along with it he sent me the WHO recommendations so I got so involved. The blue version was so boring, but later on he begun to take us through and oh… it seemed real and initially I was un comfortable but over time it got better, it seems now real.

**I: When you were starting you mentioned it helps in identifying the problem quickly. Why is that important as far as labour monitoring is concerned.**

R: For us in obstetrics, everything well, sometimes we may not say that everything is timed but there are some things which if they happen, you need to act fast, in fact they shouldn’t happen, good enough there are things that can be seen as warning signs. If you ignore them then you end up with conditions, we call evidence of neglected labour. It means things have not happened out of the blue; an obstructed labour does not just happen. It means that the warning signs were there but someone didn’t make sense of them and they continued down the sequalae into obstructed labour, then the baby is dead. The mother’s bladder already has a hole. So, for me I see intrapartum monitoring generous enough in terms of time for that person who knows what he or she is doing with the mother, who knows what can go wrong, it generous with time with the number of warning signs, which thankfully the LCG has called the alert signs. And for it tells you if you get this alert, you must address it and it has so many of them, you get me. Not just about the cervix, not just about the contractions as compare to the partograph. For it also has things to do with the woman herself, things to do with the baby, many of them. Things to do with supportive care which if you just take carelessly, they will mess up your intra partum care, labour and child birth process. So, I think the warning signs have been the big thing to take in as far as the LCG is concerned

**I: Wao, that is interesting. So, what was wrong with the partogram. Didn’t it capture these alerts**

The partograph had an alert line and action line which are 4 hours apart. So, for it, what was the alert about, on the partograph it was about cervical dilation. When you are dilating slowly, then the plot will cross from the alert line to action line and that is all. People would wait to reach the action line to act. You would find partographs and they add another one and another one or they say on this partograph the cervical dilatation is not increasing…its prolonged labour, lets send for C-section. So, they would over operate women even when it wasn’t necessary. There were 3 boxes showing strength of contractions…less than 20 seconds, 20-40 seconds and over 40 seconds, the frequency you would shade it, nothing else but with this LCG they tell you its not just about the contractions and cervical dilatation, its about moulding, caput formation, fetal position, fetal heart rate decelerations (if you have the CTG) and not just the fetal heart rate in the partograph. All these parameters contribute to the diagnosis and so help to form a more informed decision. Like I told you, its not just about the cervical dilatation and contraction.

**I: How unique is the design of this new LCG from the old partograph, design, layout etc**

R: The partograph had 5 parts, this has 7 but ours here has the eight parts that records the outcome of labour. The partograph had 5 parts so they are different and it had no where the HCP would be held responsible so anyone would put X or O and walk away with it even if it were placed wrongly but this new LCG has a place to write your initial but this one has a place dedicated to it, in addition to documenting your plan, presence of companion, fluids take, shared decision and so owning up your outcomes. The partograph didn’t have a record of monitoring second stage of labour yet a lot of things can go wrong in this critical stage for the mother and the baby. The time of pushing was never recorded but now it can be recorded. In the old partograph whenever the mother reached 10cm, then you wait to receive the baby even if it took 3 hours there was no record of the events in between 10cm and delivery

**I: Is there any component that you find included yet it’s not necessary**

R: I think the fetal heart rate decelerations which is necessary but most labour suites in Uganda lack CTG machines and where they are, HCPs are not comfortable with interpretation. Urine monitoring for protein and acetone is important but there is no where to record the volume as it was on the partogram. These 2 areas are rarely put into use

**I: Is there any important parameter that is missing in your view**

R: I think its comprehensive. I think adding the part for recording the outcome of labour like it was in the partogram has made it comprehensive 23:13

**CL004: Adult Clinician, Medical officer and Senior House Officer, LCG trainer**

**I: Thank you for giving us your time to participate in this study. We want just to ask you your experience, personal opinion with this new LCG from the time it was introduced up to now, how do you feel about it**

R: Ok, the LCG from the time it was introduced, at first it was a bit abstract. When we talked about it, my mentor shared with me, the soft copies. It looked like too far away from the reality. It looked so confiscated, so hard to use, you know. and I imagined how it is possible to use it but then as I later on got mentorship, I started getting involved in the webinars and eventually I started training others, then I started using it myself, it looked like when you do not use it you are doing a disservice to your mothers. Initially questions were very many from my colleagues who didn’t have as much as much exposure to the LCG as mine, some of them had fears of saying … when do you act, that the LCG doesn’t have the action line, so when do you act. Then they were saying .. surely were are going to loose more mothers and of course babies but I had to keep coming in and letting them know that actually the reverse is true because with this LCG you easily get the danger signs, we call them alert signs and you were expected to address every single alert as it comes and you are expected not to just look at cervical dilatation and the action lines like it was in the partograph because this mother you are taking care of is not just the cervix and the uterus that is contracting … loud laugh. So, my experience is that this tool if well utilized puts the whole mother picture, it attends to the mother holistically. Its looks at whether this mother has an attendant or labor companion, is this mother taking in oral fluids… you keep telling them to drink tea but are you sure the mother is drinking? Or its just you talking to her. These things were never on the partograph. Caring for the mother is just beyond the cervix that is dilating. So, for me my experience so far is that the LCG is so rich, is intensive and actually looks at the mother in totality.

**I: So, it is holistic**

R: Ya, that’s what I would say

**I: How easy was it for you to learn it**

R: Actually, it was easy. At first my mentor shared it with me and encouraged me to read about it. I looked at them, well, I was more interested in ither things, because along with it he sent me the WHO recommendations so I got so involved. The blue version was so boring, but later on he begun to take us through and oh… it seemed real and initially I was un comfortable but over time it got better, it seems now real.

**I: When you were starting you mentioned it helps in identifying the problem quickly. Why is that important as far as labour monitoring is concerned.**

R: For us in obstetrics, everything well, sometimes we may not say that everything is timed but there are some things which if they happen, you need to act fast, in fact they shouldn’t happen, good enough there are things that can be seen as warning signs. If you ignore them then you end up with conditions, we call evidence of neglected labour. It means things have not happened out of the blue; an obstructed labour does not just happen. It means that the warning signs were there but someone didn’t make sense of them and they continued down the sequalae into obstructed labour, then the baby is dead. The mother’s bladder already has a hole. So, for me I see intrapartum monitoring generous enough in terms of time for that person who knows what he or she is doing with the mother, who knows what can go wrong, it generous with time with the number of warning signs, which thankfully the LCG has called the alert signs. And for it tells you if you get this alert, you must address it and it has so many of them, you get me. Not just about the cervix, not just about the contractions as compare to the partograph. For it also has things to do with the woman herself, things to do with the baby, many of them. Things to do with supportive care which if you just take carelessly, they will mess up your intra partum care, labour and child birth process. So, I think the warning signs have been the big thing to take in as far as the LCG is concerned

**I: Wao, that is interesting. So, what was wrong with the partogram. Didn’t it capture these alerts**

The partograph had an alert line and action line which are 4 hours apart. So, for it, what was the alert about, on the partograph it was about cervical dilation. When you are dilating slowly, then the plot will cross from the alert line to action line and that is all. People would wait to reach the action line to act. You would find partographs and they add another one and another one or they say on this partograph the cervical dilatation is not increasing…its prolonged labour, lets send for C-section. So, they would over operate women even when it wasn’t necessary. There were 3 boxes showing strength of contractions…less than 20 seconds, 20-40 seconds and over 40 seconds, the frequency you would shade it, nothing else but with this LCG they tell you its not just about the contractions and cervical dilatation, its about moulding, caput formation, fetal position, fetal heart rate decelerations (if you have the CTG) and not just the fetal heart rate in the partograph. All these parameters contribute to the diagnosis and so help to form a more informed decision. Like I told you, its not just about the cervical dilatation and contraction.

**I: How unique is the design of this new LCG from the old partograph, design, layout etc**

R: The partograph had 5 parts, this has 7 but ours here has the eight parts that records the outcome of labour. The partograph had 5 parts so they are different and it had no where the HCP would be held responsible so anyone would put X or O and walk away with it even if it were placed wrongly but this new LCG has a place to write your initial but this one has a place dedicated to it, in addition to documenting your plan, presence of companion, fluids take, shared decision and so owning up your outcomes. The partograph didn’t have a record of monitoring second stage of labour yet a lot of things can go wrong in this critical stage for the mother and the baby. The time of pushing was never recorded but now it can be recorded. In the old partograph whenever the mother reached 10cm, then you wait to receive the baby even if it took 3 hours there was no record of the events in between 10cm and delivery

**I: Is there any component that you find included yet it’s not necessary**

R: I think the fetal heart rate decelerations which is necessary but most labour suites in Uganda lack CTG machines and where they are, HCPs are not comfortable with interpretation. Urine monitoring for protein and acetone is important but there is no where to record the volume as it was on the partogram. These 2 areas are rarely put into use

**I: Is there any important parameter that is missing in your view**

R: I think its comprehensive. I think adding the part for recording the outcome of labour like it was in the partogram has made it comprehensive 23:13

**WHO01**

**I: WT**

**Transcriber: KP**

**WHO01 is a female medical officer a midwifery tutor, to her the LCG is a modified partograph because it has the same parameters with the partograph however the starting points are different. She affirms that there a need to explain to the health workers where the LCG is coming from for them to accept it well. For the successful adoption and implementation of the LCG, she suggests that there is need for proper designing of the LCG tool, the need for proper training of all the users, supervision of the implementation as well as availability of the required resources. To her this is all about maternal health and the sustainability mothers and their babies through proper labor monitoring thus she assumes all users of the LCG tool will welcome it.**

**I: We are trying to learn about the implementation of the new labor care guide (LCG) that has been suggested by the World Health Organization (WHO) so in this interview we would like to understand the different factors that can facilitate the implementation of the new LCG in Uganda. Now from your experience as an implementer what are some of those factors do you think should be in place for the successful implementation? What are your thoughts when you compare the new LCG and the old partograph in relation to labor care monitoring?**

**R: The LCG is monitoring the same things we have always monitored in labor using the partograph, the parameters are the same we want to understand how the mother and the baby are progressing in labor and we are monitoring the vital signs, we monitor the features that shows us that the woman is progressing in labor and that the fetus is alive while keeping the normal heart rate.**

**Although the starting point is now differing the person who is monitoring the mother must be well trained for them to be able to understand that when the heart rate of the baby is moving like this, these are the normal limits. Even for the mother one must know that this is how the uterine contractions must be, this is how the dilatation must be. So that principle has been sustained and it’s even stronger now because the other elements which are measuring whether we are being respectful and whether we are explaining what we are doing as health workers. Those are the things in which many health workers have not been oriented and not appreciating that this time the need for the engagement with the mother and the need for her to score you in relation to the work you have done is also part and partial of the work you have done. That area is new and we need to teach it. And we need to ensure that every health worker, the doctor and the mid wife understand what it entails and why it’s there.**

**I think that even as we start we need to make people understand that this is based on a global survey because results are known and they should be disseminated preservice and in service so that the health workers can understand where we are coming from. We labored with the partograph for so many years. And even that smaller component still we having many issues with complying and yet where expected to comply is the normal clinical practice in which we have been trained for so many years all of us. Now this one that related to attitude and behavior in new, not completely new but honestly during our training we never we into details as to why it should be there so we must start health workers from that understanding that we are opening an area which we need to explain with its involved elements otherwise they can’t second it very well.**

**So this is the LCG is some complicated tool thus we must understand that it’s going to take some time in the orientation of the basic elements. And the DHO is very serious when it’s adapting such guidelines in countries and it usually wants to address the local situation by getting information from different levels to understand how we can move better forward.**

**So it’s a progress even the design is the process I mean how the LCG will look like in Uganda, I don’t know the level we are at but the normal process would be knowing what we currently have, knowing what need to add on and how do we move as a team and the design would a contribution of different practitioners and I think this needs to be done.**

**I also think this process would also begin from an external view and it would also be good to understand if there are1 or 2 countries in Africa that have tried to do the adaptation and what did they find or what did they try to do differently. Similarly our process now begins from the university which is okay but it needs to take on a consensus perspective and the design. The design is so important because if you see what we are currently dealing with, it has not come easy. We have had to teach each area in details and it is still one of the poorly performing areas even after all these years.**

**For us now the LCG is an exciting piece which is a second piece, we struggling with the first piece but we never gave up because we know that this is an important area and we shall definitely be happy to be part and partial of this pilot. Therefore, if we can get the designs done because already we have something but this design is very compacted because I have to put on glasses which is why the design is so critical. Everyone should be able to use it without having to squeeze things thus the design should be taken into consideration and even the instructions. So all I am saying that we need to engage the people in the university and also the public health specialist users in as far as design is concerned because we have seen people having many complaints, they have to run around labor ward trying to figure things out. The university training is different from the implementation so you must make sure that the tool fits the users easily, it has to be readable, and the instructions must be clear and attractive.**

**But we are all ready to go after making sure that the most common users who are the midwives are conversant and comfortable with this piece. We have many interested parties who can look at this piece and design it to fit where Uganda is now.**

**I: Is there any other thing that you would want to see?**

**R: The beginning is the design to make sure that everyone is able to use it, there is also a need to design a training manual for health workers something can help in explaining the elements. It should be easy for everyone who looks at the tool to find where the issue is.**

**We all believe that this is maternal health and this is what will sustain women and babies we need to monitor them well when they are with us then we have to give it time and make sure that all people trained the same way. All people need should be trained on how to use it systematically so that if I find a midwife in Mbarara and another one in Soroti, they will both be saying the same things and plotting in the same way.**

**The challenge we are met with is that people seem to say different things and you wonder where they are coming from, that’s why we need to standardize the tool and have a guideline that will ease the implementation.**

**I: Given all these you have talked about, do you think Uganda is already to implement the LCG?**

**R: Yes, but we have to put in a lot of energy and make sure that we train people so that they know how important it is for their own quality of care and also for the outcomes for the mother and the baby, and why it is different. And this will take time, because we worked with a simpler tool for so many years but we still see issues. It won’t be easier but if we all agree and begin off well we shall be able to move because right now we have so many midwifery tutors, by the time we came up with this we were very few less than 30 in the whole country but there is a big force that can move this quickly, we have a good environment so we just need to come together quickly, commit ourselves and see how to move forward because we have to manage a transition from the partograph now. This will be easy if we have a meeting as personnel and conclude that this is what we are moving forward with, we need to have competent trainers who will take it through.**

**I: Doesn’t that raise logistical and financial issues?!**

**R: It does, it will always require some resources but we are also not void of resources as long as there is some concession in the national ministry, we can the leadership, the students and that make the effort so we have some resources, Dr. Mugyenyi a resource already so if we get the process right from the beginning I do think that there are some partners who can support the cause. It’s a journey but I think we must undertake it and I don’t think there is anybody who is doubtful because everyone is now talking about respectful maternity care that’s why I am convinced that we can move this forward as a country.**

**I: For example when we are reading literature about the partograph, you find that people are trained but coming down to implementation, there are those challenges that come in place and you feel that they are failing to use the partograph!**

**R: Those are the things we will have to put into consideration now and already the central ministry and the regional maternity systems are battling with the accountability. Now for the LCG there must be a champion on the ground in that maternity unit to move the process of using this LCG properly. In my training as a medical officer I was happy to be under the supervision of an OBGYN who said that my young doctor in this labor ward we shall know other people she would supervise you and make sure that your learning. This what is required, the need for someone who will solely be dedicated to championing this work and make sure that every maternity labor ward is serious on monitoring of mothers.**

**The doctors will have pick a champion, the in charges will also pick theirs, and they will be accountable for each mother who enters that’s why I was telling you in the beginning that people must be knowledgeable enough to know when to start and who to start on the LCG or not.**

**A number of things will also have to start gradually because change can’t be so sudden. I have described to you the maternity ward where I took a lot of interest in the partograph, when I left that place that’s when I started moving in the country to make sure that a partograph is being taught and the users are committed and there is a person in charge of making sure that its done, you can’t just leave it but at the moment we have left it. All the doctors attached to these health centers and the midwives must be properly trained so that they can also train others, the midwifery tutors also need to be trained so that we can also push it.**

**I: Considering like health centers III now I realize the importance of having those champions but you have talked about the fact that everything has to change!**

**R: For example when a woman enters she must have a folder and the LCG has to be in that folder so that whoever enters has where to document, the doctor and the midwife. Currently we see a number of mothers in labor with their own exercise books that’s where they are documenting you can’t do this with exercise books so at that level we will have to advocate for the availability of the correct stationery, training and proper record keeping thus we need box files to make sure that we can compile and keep reviewing. In some health centers IV that’s still maintained because that’s the strategy, the doctor and the midwife continuously check their weekly performance, they check their challenges and progress so that there is an opportunity for change but if are not motivated they won’t be able to do this.**

**I: Motivation is very important!**

**R: Yes, motivation and accountability.**

**I: In an environment where health workers don’t have salary, don’t you think that might be chaotic?**

**R: I think it’s chaotic.**

**I: As we conclude, what in store from WHO the originators of this LCG to make sure that the adoption and the implementation is done thoroughly in Uganda?**

**R: For use we wait for the official request from the ministry of Health and to show the willingness that they want to follow the process of the normal adaptation and once this letter is received from the central ministry going to the world it raises the colleagues who can support a process of this nature, sometimes they raise resources for meetings to be able to do this in partnership with the stake holders in the country. There must be something that shows that a country is ready for the adaption and it is willingly requesting for the technical support.**

**I: So that technical support is in which form?**

**R: First WHO identifies technical people who will come and support the process then it comes to check the process and all that is resources, WHO usually requests a country to look for other partners.**

**I: Do you have anything that you want to say in addition to what we have been discussing?**

**R: We will face some challenges because this is also a partograph though modified but they are the same principles but we must be able to determine that we move this forward and devise means and methods of empowering the teams that matter to be able to do that. This includes bringing together stake holders, and a lot of discussions because it must be country owned and used both in public and private health facilities. Considering the energies this should be able to move.**

**I: Thank you so much**

**Transcript CL005**

**I: Tell me your experience about the new LCG which is in the process of being rolled out to monitor labour**

**R:** Thank you so much. We have been using the LCG here at MRRH. I have personally used it and participated in training under graduate students and other health workers on its use. My experience has been good. I believe it’s a better tool much better than the partograph which we have used for a long time. The partograph was making us intervene when it is too early, before needing to intervene. It’s a very nice tool that is supposed to help us modify the care we give to mothers in labour

**I: Generally, how do you compare the new LCG and partograph**

R: LCG is surely a better tool because it has got what we call the alert column that comes with a very good feature where you as a health worker monitoring labour you get an alert at every point in that when a mother in labour comes alone, without a husband or any other attendant it is an alert, if I find the mother sleeping on her back, at that time it’s an alert that she is not in the right position favorable for labour progress. If I find when the fetal heart is not at this level, it’s an alert, if the contractions are too few or even too many, it’s an alert. So, it comes with the alert column that is a very great feature which if every health worker responded timely/appropriately would solve the needs of every mother on labour. This new embedded LCG feature is contrary to what the partograph had. It had the alert line and action lines but largely on the cervical dilatation, yet anything can go bad including the blood pressure, urine protein etc. really the partograph didn’t have an alert and action on abnormality of every parameter/measurement, meaning that if the HCP doesn’t critically measure, record and interpret each parameter, then they would leave a mother with a danger sign that is un recognized, but for the LCG, there is an alert for every observation and therefore no mother with a danger sign will be un recognized or unattended to.

**I: Is there any other thing that you like about it?**

R: the other thing I like about the LCG is that it helps monitor and record events in the second stage of labour including time spent in second stage. It also documents the time the mother starts pushing and therefore I can accurately tell the duration the mother has been pushing, for 30 minutes or 1 hour making it easy to objectively make the diagnosis of prolonged second stage of labour. These attributes never existed on the partograph. I think of course there are other features which are largely the same, that we embrace.

**I: What about the challenge. Did you face any challenge while using this new tool?**

R: Personally, I have not got any challenge. Once you have the right training and you know what you should fill at each section, you do not meet any challenges.

**I: How easy was it for you to learn this tool. Was it easy or it was difficult**

R: Initially when I heard about it, I really took interest to download the WHO manual and read it. It is well summarized, and if you read it you do not find it hard to fill the LCG.

**I: What motivated you to download the WHO LCG manual, to read through. Ehat was that underlying force?**

R: *…..long laugh…* I don’t really know what motivated me but…just looking at what we were experiencing and the key thing behind the change, looking at how the partograph was designed, the alert line and the action line, there were certain assumptions that actually in real life do not apply like expecting that every mother’s cervix would dilate at 1cm/hour, then you get a mother who is seemingly fine, then you examine them after 4 hours, you find actually they are not far away from what they had. Actually, the partograph assumed that if a mother was 4 cm now, then you examine them after 4 hours she should actually be 8cm yet in real life this is not true for all mothers. You get these mothers whom you examine and they are still 4 or 5cm but are relatively stable. Whenever you give them time, they progress and deliver normally without any interventions. Then I realized that if there is a tool addressing that I should be interested in it and I read about it

**I: That’s impressive. What do you think about the design. Do you like its design? And the lay out. What do you say about the design of the LCG, wording etc.**

R: I think some of the words are a bit small but can really be read. Maybe a paper larger than size A4 but generally speaking it can be useable as is. Maybe putting it on hard paper, becomes an official document and once I have the LCG, actually what I forgot to tell you is that if you look at how it is made, I do not think HCP should be burdened with writing a lot of notes in the patient’s file. The LCG has got everything that actually you would want to write in the notes. If I have filled my LCG very well and put my initials at the end which the partograph actually didn’t have, there was no ownership. You wouldn’t know who has filled it, I do not need to go back and write the patient’s blood pressure in the file, the contractions, the amniotic fluid…every other thing yet they can be all recorded on the LCG. I think this is something perhaps we need to address at all levels that the HCP does not need to write both on the LCG and the patient notes. Otherwise, people will look at it as extra workload and at the end of the day they will not use it.

**I: So based on the normal patient file, you mean if this LCG is properly utilized, it captures all that data**

R: Almost all the information that we need to write about the patient who is in labour can be captured on the LCG without necessarily writing in the patients file which would create extra workload and therefore reduce utilization of the tool.

**I: are there any modifications that you would suggest on this LCG depending on the sections? Any words or redundancies or repetitions? Is there any changes that you recommend for this?**

R: there is no repetition, of course there is an issue on the part of recording the fetal heart rate baseline and decelerations, I understand that this tool is to be used for all levels of health care facilities. That section would need CTG machines for monitoring and recording these decelerations. These CTG machines are not available in lower facilities like HC III and IV where labour monitoring occurs, even in general hospitals and regional referral hospitals I do not think every mother is monitored using CTG. I think HCPs need to be told that the tool is designed for every mother in all health facility levels including those with CTG so for facilities without CTG, this section can be left blank. We actually do not have to remove this parameter because it will apply to that mother where you actually need to do CTG.

**I: What do you think about the fact that labour monitoring using LCG begins at 5 cm, yet in the partograph it begun at 4 cm. What do you think about that change in regard to labour monitoring**

R: Yaa, 5cm is ideal. I know and I have read that it followed evidence-based research findings that showed that actually labor does not always progress very well until the mother is about 5 to 6 cm. I also know that there are some countries which actually use 6 cm as their cut off for active labour. I think 5cm is a good thing compared to 4cm because if I look at what we were doing with the partograph, actually those we would start monitoring labour at 4cm are the mothers who would be tagged as prolonged labour because at 4 cm the contractions are still few, the cervix may not be well effaced and therefore you may not expect this mother to progress very well. When you start monitoring at 4cm chances are that you with find the mother still 4 cm after 4 hours and you start labeling them prolonged labour yet everything is still okay.

**I: That’s amazing. What changes have come with the introduction of this new LCG in this facility. Have you observed any changes that have been brought about by this LCG?**

R: I haven’t gone into details of documenting the changes, but I think for people who are passionate about using it, I have heard them say it’s a better tool. I believe it should be able to reduce un necessary C-sections and perhaps do timely C-section for those women who actually need it. You know the old partograph had an action line and alert line and so every time the plot would cross the action line someone be compelled to do an action and I can assure you most actions HCPs would be to put oxytocin for augmentation of labour or d a caesarean section. So you would find mothers on Oxytocin before they need it or they get a C-section before they actually need it. I actually already said that somebody would cross an action line before they need the action because of how the partograph was designed, expecting you to dilate at 1cm per hour which is not practical for all mothers

**I: That’s interesting. How did you feel when you were told you would begin using the new tool compared to the partograph. Some times change in not being flexible. Sometimes you are used to team ways…how did you feel about it**

R: Personally, I welcomed the idea.

**I: What do you have a s a facility that is I place to make labor monitoring better, the things that are around that facilitate labour monitoring**

R: The team work that we have as residents, that actually we have become responsible. That if someone monitors labour, the next person who come after you he is going to be asking you how this mother has been progressing. So if you have not monitored, then you cannot respond.

**I: and what are those things that are lacking to make labour monitoring better**

R: Maybe they may not be lacking but we definitely have many patients here needing labour monitoring at the same time, which interferes with timely observation and recording on the LCG

**I: What do you mean by interfering**

R: If for example you are supposed to listen to the fetal heart rate every 30 minutes (even with the old partograph) and if I have 5 mothers in labour and I have another emergency, I may not be able to adhere to that recommendation. You find I am listening to the fetal heart every one hour or 2 yet actually I should have listened to it every 30 minutes.

**I: Which effects ….**

R: Yes, that may have an effect because if the fetal heart changes, you may detect it late which has consequences.

**I: That’s good. Apart from training and mentorship that you talked about, what other things that were put in place to motivate you people, the staff, the clinicians to begin using the new LCG**

R: I think the time they said we were going to start using the LCG, they stopped printing the partograph papers so that if you must monitor labour, the only tool that was available was the LCG rather than having both tools and people tend to use what they were used to.

**I: Who prints those LCGs**

R: The hospital and sometimes working with the university

**I: Did you have logistical support like pens, anything to facilitate your usage the new LCG**

R: No.

**I: Do you thing you may have them, because some one says that there is a part where you are required to use like a red pen when you detect the alert? Do you use the red pen always??**

R: I have not used the red pen always but I believe that the way it is designed, even if you have not used it, the whole thing is that I am circling all alerts and it is not possible that you will not see where the circle is. So even the times I have not used the red pen, I don’t think I have got any challenges.

**I: What do other people like the workmates when they see you using this new LCG, what do they say, what do they feel**

R: By the way here we are now used, the ones out there is eager to see this rolled out

**I: Now you as a trainer of trainees, as we conclude, what are some of the take home lessons, that if you are going to implement this LCG tool these are the things that you should bear in mind, that as you train people to use this tool, you said that you trained undergraduates.**

R: I think the undergraduates and HCP at all levels need to know that LCG is a better tool and that it doesn’t create extra work load and should embrace it. My prayer is that the ministry of health will roll this tool in all facilities as soon as it is possible

**I: Why, why should they roll out as soon as possible**

R: We definitely should not have double standards. Some facilities are using the partograph and others are using the LCG. I actually forgot to tell you that the LCG tool is a WHO tool of 2018 so we are five years late, we should have started using the LCG five years ago

**I: Ok, thank you so much for this wonderful discussion**

**CL004: Adult Clinician, Medical officer and Senior House Officer, LCG trainer**

**I: Thank you for giving us your time to participate in this study. We want just to ask you your experience, personal opinion with this new LCG from the time it was introduced up to now, how do you feel about it**

R: Ok, the LCG from the time it was introduced, at first it was a bit abstract. When we talked about it, my mentor shared with me, the soft copies. It looked like too far away from the reality. It looked so confiscated, so hard to use, you know. and I imagined how it is possible to use it but then as I later on got mentorship, I started getting involved in the webinars and eventually I started training others, then I started using it myself, it looked like when you do not use it you are doing a disservice to your mothers. Initially questions were very many from my colleagues who didn’t have as much as much exposure to the LCG as mine, some of them had fears of saying … when do you act, that the LCG doesn’t have the action line, so when do you act. Then they were saying .. surely were are going to loose more mothers and of course babies but I had to keep coming in and letting them know that actually the reverse is true because with this LCG you easily get the danger signs, we call them alert signs and you were expected to address every single alert as it comes and you are expected not to just look at cervical dilatation and the action lines like it was in the partograph because this mother you are taking care of is not just the cervix and the uterus that is contracting … loud laugh. So, my experience is that this tool if well utilized puts the whole mother picture, it attends to the mother holistically. Its looks at whether this mother has an attendant or labor companion, is this mother taking in oral fluids… you keep telling them to drink tea but are you sure the mother is drinking? Or its just you talking to her. These things were never on the partograph. Caring for the mother is just beyond the cervix that is dilating. So, for me my experience so far is that the LCG is so rich, is intensive and actually looks at the mother in totality.

**I: So, it is holistic**

R: Ya, that’s what I would say

**I: How easy was it for you to learn it**

R: Actually, it was easy. At first my mentor shared it with me and encouraged me to read about it. I looked at them, well, I was more interested in ither things, because along with it he sent me the WHO recommendations so I got so involved. The blue version was so boring, but later on he begun to take us through and oh… it seemed real and initially I was un comfortable but over time it got better, it seems now real.

**I: When you were starting you mentioned it helps in identifying the problem quickly. Why is that important as far as labour monitoring is concerned.**

R: For us in obstetrics, everything well, sometimes we may not say that everything is timed but there are some things which if they happen, you need to act fast, in fact they shouldn’t happen, good enough there are things that can be seen as warning signs. If you ignore them then you end up with conditions, we call evidence of neglected labour. It means things have not happened out of the blue; an obstructed labour does not just happen. It means that the warning signs were there but someone didn’t make sense of them and they continued down the sequalae into obstructed labour, then the baby is dead. The mother’s bladder already has a hole. So, for me I see intrapartum monitoring generous enough in terms of time for that person who knows what he or she is doing with the mother, who knows what can go wrong, it generous with time with the number of warning signs, which thankfully the LCG has called the alert signs. And for it tells you if you get this alert, you must address it and it has so many of them, you get me. Not just about the cervix, not just about the contractions as compare to the partograph. For it also has things to do with the woman herself, things to do with the baby, many of them. Things to do with supportive care which if you just take carelessly, they will mess up your intra partum care, labour and child birth process. So, I think the warning signs have been the big thing to take in as far as the LCG is concerned

**I: Wao, that is interesting. So, what was wrong with the partogram. Didn’t it capture these alerts**

The partograph had an alert line and action line which are 4 hours apart. So, for it, what was the alert about, on the partograph it was about cervical dilation. When you are dilating slowly, then the plot will cross from the alert line to action line and that is all. People would wait to reach the action line to act. You would find partographs and they add another one and another one or they say on this partograph the cervical dilatation is not increasing…its prolonged labour, lets send for C-section. So, they would over operate women even when it wasn’t necessary. There were 3 boxes showing strength of contractions…less than 20 seconds, 20-40 seconds and over 40 seconds, the frequency you would shade it, nothing else but with this LCG they tell you its not just about the contractions and cervical dilatation, its about moulding, caput formation, fetal position, fetal heart rate decelerations (if you have the CTG) and not just the fetal heart rate in the partograph. All these parameters contribute to the diagnosis and so help to form a more informed decision. Like I told you, its not just about the cervical dilatation and contraction.

**I: How unique is the design of this new LCG from the old partograph, design, layout etc**

R: The partograph had 5 parts, this has 7 but ours here has the eight parts that records the outcome of labour. The partograph had 5 parts so they are different and it had no where the HCP would be held responsible so anyone would put X or O and walk away with it even if it were placed wrongly but this new LCG has a place to write your initial but this one has a place dedicated to it, in addition to documenting your plan, presence of companion, fluids take, shared decision and so owning up your outcomes. The partograph didn’t have a record of monitoring second stage of labour yet a lot of things can go wrong in this critical stage for the mother and the baby. The time of pushing was never recorded but now it can be recorded. In the old partograph whenever the mother reached 10cm, then you wait to receive the baby even if it took 3 hours there was no record of the events in between 10cm and delivery

**I: Is there any component that you find included yet it’s not necessary**

R: I think the fetal heart rate decelerations which is necessary but most labour suites in Uganda lack CTG machines and where they are, HCPs are not comfortable with interpretation. Urine monitoring for protein and acetone is important but there is no where to record the volume as it was on the partogram. These 2 areas are rarely put into use

**I: Is there any important parameter that is missing in your view**

R: I think its comprehensive. I think adding the part for recording the outcome of labour like it was in the partogram has made it comprehensive 23:13

*22 minutes*

**Participant ID:                                  CL007**

**I:                                                        WT**

**Transcriber:                                     KP**

*CL007 is a male gynecologist working with Mbarara Regional referral Hospital to him the LCG is an exciting tool that gives better and satisfying experience to mothers in labor, he states that it helps health workers in labor suit to make more informed decisions of women who aren’t progressing well in labor which actually reduces on the number of C-sections performed as a result of prolonged labor. However he states that in cases where all health facilities are not using the LCG in labor monitoring, there could be a challenges whenever there is a referral of a mother from a hospital that’s not using the LCG to the one using it as there is no uniformity in the system of communication. Also we wishes the LCG would be provided in form of a hard paper for proper handling.*

*I: How do you feel about this new Labor care guide (LCG) tool?*

R: To me the LCG is an exciting tool and I think it will revolutionaries the neo care, the intrapartum care of women. Right from internship I had for long realized that women actually spend longer in labor than the partograph was actually dictating so amid wife would call you that the mother they are monitoring has crossed the action line and then you go and give another 2-3 hours. You would come and examine her, find that she is okay and giver another 2 hrs because you don’t find any reason to operate her and at the end of the day may be after 6 hours she delivers normally and that made me think that actually the partograph was for women who progress very fast in labor but for women who are very slow, you will end up operating them because they have crossed the action line with no clear reason. So for me the LCG is a good tool.

*I: So how easy was it for you to learn to use it?*

R: I don’t know whether it’s because I had tried to read about it before because the tool came up a bit earlier and by the time we received it, it wasn’t difficult. I think it’s very easy to follow, the LCG it’s self actually guides because it’s a matter of ticking boxes. You can see that the woman has spent this period of time at this level of centimeters so I think it’s easy.

*I: How do you compare the partograph and this LCG in regards to monitoring fetal or maternal wellbeing?*

R: The LCG is a very comprehensive tool and for me and in fact for me my practice I think that I don’t have to write any notes unless it is really important that I must record something extra which is going to be just a message otherwise the woman who is progressing well in labor will have all their records on the labor care guide and will be complete as opposed to the partograph. The partograph only had things to do with the cervical dilation and the heart rate so really it wouldn’t give you that detail of do you have a companion, are you mobile, what’s your position because all these have an impact on the outcome of labor because if the mother is not supported you don’t expect the things we call for cleared. The last things about psychological preparedness which is actually more attached to the companions of the mothers because they are the ones who can help them remain positive so I think in terms of recording it is more comprehensive it gives more clearly clinical picture of what is happening.

*I: Doesn’t that comprehensiveness make it challenging?*

R: No I just had a training with the midwives on how to use the LCG and some of the things I can tell you we realized is that the LCG eliminates that need to write extra notes, while using the partograph you would have to write extra notes in the file which is not necessary with the labor care guide, yes it is comprehensive but it ensures completeness of notes and eliminates that extra time. You would have to fill the partograph and then look for the file and start writing.

*I: What’s your comment on its designing/he layout, do you have any concerns with the design or is there anything you think should be added?*

R: The first time this was introduced we didn’t have a space to record the outcomes it was only ending at the second stage of labor, even if you look the World health’s original chart it doesn’t have the space for outcomes, what we have done at the referral with the help of Dr. Mugyenyi being the specialist in the labor suit and admission, he helped us to add that part where you need to write the outcomes and of course the immediate care given to the mother and the 3^rd^ stage so I think what is necessary is adding that part that misses which is the outcome of labor, the management of 3^rd^ stage and the immediate care given to the new born. May be for example let’s say you have given an episiotomy there should be some space to put my notes that this happened and I have done this, if that is added it makes it a complete file because now the antenatal card attached which provided all the antenatal history and the labor care guide attached which gives all the intrapartum care and the postpartum care given within the immediate period which makes it a complete file.

*I: So it reduces on filing?*

R: Yes it reduces on filing, you see the more papers you have the more difficult it for health workers to fill and also its very easy to keep the first time I saw partographs they were on hard papers manila like, I wish the LCG was also like that on a hard paper. It eliminates a need for files, and extra papers.

*I: Why do you want to be on a hard paper?*

R: Because of handling you see we are in labor suit, I am monitoring a mother I need to wash my hands then come back and record and you know hand driers and towels are very few in our labor suits so a drop of water on just a sheet of paper can distort it and even the writings.

*I: Is there any challenge that you have encountered while using this LCG?*

R: I have given the LCG a lot of credit and I think it deserves it but I have a challenge with it, you see the partograph used to direct anyone whether you have gone to school or not as long as you had specific training in midwifery, you could see that now we have crossed the action line/ alert line so I need to act. The LCG doesn’t do that, it gives you alerts at almost every point, you have no companion that’s an alert, the fetal heart is low its shows an alert, the contractions are low that’s an alert of course in the partograph that wasn’t there it wouldn’t tell the contractions are too many or too less. With the LCG these alerts are there which is a good thing but it is the user to make that interpretation. It’s a good tool which needs good training, it requires the user the understand the basics of midwifery and obstetric care, that’s my only challenge with it. If I have an old midwife, I have worked in the government setting so we have midwives who come from health centre II after 5 year and that person has forgotten everything she is as good as a 1^st^ year student so that’s where I have an issue but I think if we can have some good training I think there wouldn’t be a problem.

*I: Is there any other challenge apart from that?*

R: I don’t think there is any other challenge I have personally observed in use. However for the system the challenges are there, I have already told that we have trained some midwives so some people are using it others are not using it so I was in Rwekubo where I was told that the they don’t use the LCG about a week ago now if a woman is referred from Nakivale where they are using the LCG (which is a good thing), now when the woman is referred then it means that there will be some challenges because the system of communication is not the same so I am looking at the time when the LCG will be rolled up so that we can have the same platform of communication.

*I: What changes have come in place with the introduction of this LCG?*

R: We now all recognize and agree as a profession that actually labor doesn’t progress by 1cm per hr that labor may be quicker in some women and may be slower in some women that is now the major changes that we can now allow women to stay a bit longer in labor because we know as long as the mother is okay and the fetus is okay we don’t have to operate that woman because they have simply crossed an action line. It helps us also to interpret if a woman had spent longer at a certain point for example in the progress of labor then it helps us to find out the problem and solutions.

So the change is that we can now comfortably allow women to stay longer in labor as long as they are okay. Another change is that it produces a major component of respectful maternity care, a woman can now come into labor suit with her companion and it is okay no one sees that as intrusion as long as she has chosen that this is my companion then I know whom I can talk too. Sometimes women can be anxious when they are in labor especially when they are on the second stage and its good when they have someone to hold their hands or their back.

*I: Do you have some people you have heard who have not used the LCG, I mean people who are resistant to change or have you had instances when you have tried to check in the file and someone has not filled?*

R: You know change doesn’t come easily I will start with what we did a few days back, we went to Rwekubo and trained people in Rwekubo, Nakivale and Nyamuyanja but there are those who take it on and those who don’t take it on but usually it’s a question of being ashamed because people don’t feel responsible, someone just doesn’t want to do what they are supposed to do so I don’t know if that’s a challenge of the labor care guide or it’s a challenge of leadership. Even here at the referral you find that someone has monitored the patient, written on the board (we have a board where we write the mothers we monitor) and they are following up on the progress but they are not record.

*I: So that’s attributed to personal negligence or may be someone is busy?*

R: Someone not doing their work could be that may be they are too busy because at the referral you can actually be overwhelmed you find there are many women in labor so how can you monitor those, it becomes very difficult but also the resources. Looking the labor care guide you realized that for example you are supposed to be monitoring the fetal heart rate so if you have 8 women and you think about it properly it becomes very difficult because of the instrument we are using. Some of those challenges are system based but sometimes its negligence of the people who are supposed the work.

*I: What are those things that someone should bear in mind when they are going to rollout this LCG tool assuming the ministry of health wants to adopt, what are those things that are supposed to be in place to facilitate the use of this tool for monitoring of labor?*

R: To me the ministry has already adopted this tool which is a good thing may be what is remaining now is the rollout. You see everyone in the care of women hates a maternal death if we could help people understand that using the LCG helps us to make more informed decisions of women who aren’t progressing well that’s important. If people realize that using the LCG could reduce on the number of caesarian sections performed due to prolonged labor that would also be good. I feel it gives every women a very satisfactory experience, it’s really unfair for a woman to come into labor and after 6-8 hrs you tell her that you have to be operated because you have cervical dystocia and this is common when nothing has been done, the partograph wasn’t talking about those but now the LCG is talking about them so you realize that you can now reduce that burden of C-sections.

So the most important thing is to help people understand that the LCG gives a better and satisfactory experience to mothers without forgetting that a women who come into labor with their companion also get good experience.

*I: As we conclude is there any other thing you would like to tell me about this discussion?*

R: All I can say is that the LCG is a good tool that should be recommended, adopted and used and also adopted for local use whenever there are special circumstances. Most of the other things we have talked about them, I would prefer it if it was on a hard paper for proper handling.

*I: Thank you very much Dr.*

37 minutes and 46 secs

**Participant ID:                                  CL008**

**I:                                                        WT**

**Transcriber:                                     KP**

*CL008 is a female midwife at Mbarara Regional Referral Hospital who has used the LCG in labor monitoring and she appreciates the fact that with the LCG you examine a mother and act immediately unlike the partograph where you had to wait for the action line to plan, she so much in love with the idea of shared decision making that comes with using the LCG. However, she feels that there is still a challenge of knowledge gap and negative attitudes that limits the use of the LCG in labor suit and she feels these can easily be addressed by the peer to peer approach. She states that the use LCG tool is also challenged by limited beds within the hospital thus she thinks that if the government were to rollout the tool in all health facilities they have to make sure of expansion, availability of stationery and making sure that all the intended users are equipped with knowledge.*

*I: Thank you so much for accepting to give us this interview, like I said we want to understand your honest opinion regarding the use of this labor care guide so I want you to tell us your experience in using it.*

R: We are using the LCG tool to monitor mother in regards to the mother’s conditions, the feta’s condition and the labor progress and it helps us to assess the mother’s progress, the mother’s condition and the fetus condition and it helps us to plan and take action immediately. So that’s basically what I am using it for.

*I: So how do you practically do that?*

R: Practically I examine the mother and I find that the mother is 5cm and above I start plotting and fill the bio data which is the 1^st^ part, then I state the time I have examined the mother then I start plotting the labor progress and all that, then I plot the fetus condition and look out for any alerts/anything that’s is worrying. If there is any worrying situation such as deceleration/acceleration you note it somewhere, you put in red pen if not you just bold it with a blue pen. Then I go down and plot everything in case I find any abnormalities, I write a plan for example I am a midwife so I take nursing care for example if a mother has fetal distress I won’t say emergency C-section I rather put in my plan and put in some iv fluids then inform any doctor around as I monitor this mother.

*I: How do you compare this LCG to the old partograph?*

R: There are many advantages with this LCG from the partograph for example this LCG has apart where you have to assess then you plan and act and show what you have done about it but for the partograph we used to plot and finish there was no part for planning and acting. Then the partograph had the alert line and the action line so until the mother has gone to the action line we wouldn’t act but for the LCG we can’t immediately. With the partograph the mother would be there with 2 contractions at 5cm and she keeps there with her 2 contractions until the labor is prolonged she is action that’s when you say this mother has no contractions let’s do this and remember she has already spent 8 hrs. With the LCG if you find that the contractions are less for the dilation of the cervix, immediately you have to act either finding ways of stimulating or augmenting that labor so you act and plan.

Then another thing about it is that the other was entirely about a service provider but this LCG is between the client, the attendant and the health provider because you have that plan of shared decision making so you have to plan with the client and make a decision together and implement the plan together.

Then I have noticed another difference on the second part of the LCG, on the partograph there was nothing like 2^nd^ stage monitoring. If the mother is fully dilated then the 2^nd^ is done yet we have some mother who take some time in the 2^nd^ stage so it is very good because it helps us to monitor these mothers in 2^nd^ stage until they have pushed their babies safely with the partograph you would stop listening to the fetal heart when the mother was 10cm and you start telling the mother to push without caring about the monitoring what if the fetal heart has decelerated or is accelerating. So this is good because we have been losing babies because of that 2^nd^ stage not being monitored well. So those are the basic changes I have seen with this labor care guide.

*I: You have talked about shared decision making, why is that important?*

R: It is very important because for example and make a plan myself then tell this mother that I am going to augment your labor mind you she is a caesarian scar and in the beginning she has lied to me that she has never been operated because she is not aware that I am going to augment them, she knows the contractions are going to come normally and she has lied to me that she has never been operated but if I make a plan to augment this labor with an oxytocic or a stat pearls I am going to come and tell her with the attendant/companion that the labor is prolonging because you have no contractions so I would like to augment to add you more contractions. If she hears about those artificial contractions and she knows that she has a scar but she has lied to me trust me she is going to come clean about her lie because she knows the danger since they always told that you’re not supposed to get those when you have a scar. That helps you to make a better decision than making a decision that would make a mother to rapture.

Another thing, we always make a decision and tell mothers to go and walk but this mother may not be able to walk at that moment, there are mothers who get paralysis she gets contractions and she fails to walk yet I keep forcing her to walk, but if I sit with her and she tells me about her problem we can make a shared that is good for decision together so shared decision making is very important.

*I: How easy/difficulty was it for you to learn using the LCG?*

R: It was quite difficult at first I was taught by my colleague midwife and it was so challenging, I first failed to understand these abnormal alerts but with time I realized it was just easy then Dr. Mugyenyi also brought a CME and we learnt a few things which made it easier because we were used of the other partograph. You know when you are used of analogue then they bring digital things you first get challenged but I was able to understand it within a few days.

*I: Did you learn it because they had put it up for you guys to use or you’re wanted to get a skill?*

R: Personally I like learning new things, I am always eager to learn whenever they say that there is a new thing, I won’t talk for other because I have heard some people who are grumbling about it saying that they are used to using the partograph but I like learning because I know that in medicine things keep changing so if your rigid then you miss out on good things that would change quality care. So I embraced the change and I was eager to learn because I remember they brought it when I was on leave so I came to find new things and I forced those had learnt about it to teach me.

*I: You have said that at first you had some challenges, what could have been done to make sure that someone will learn to use this tool without any challenge?*

R: The best way would be training at least two midwives and doctors about it and then giving them the responsibility to teach others very well before they introduce the tool. They would offer training to everyone and even allow us practice using it before introducing it because there are still some midwives who don’t know how to use it especially those who aren’t eager to learn.

*I: In addition to that, how best can we engage those ones who seem to be resistant?*

R: I think we need to look for champions of the labor care guide, we can get 3-4 midwives they be responsible for implementing this labor care guide, they make sure that for every mid wife to step in the labor suit is able to plot the LCG, they make sure that this person learns.

*I: Do you think it’s only about not being trained or there is another challenge?*

R: There is also the issue of attitude like I have told you some people do not care, some people be like I got jobs and feel nothing else matters but for example I know how my different colleagues behave so I know how to handle them differently so it is easy for me to find ways of teaching them that’s why I talked of 3-4 mid wives and not doctors or the in charge because we are closer to those with their attitude and we can easily talk to them but if the in charge tries to teach their attitude is already in negative.

*I: So you prefer peer-peer learning?*

R: Exactly you have put it very well because I have realized that most of the people don’t care about the CME’s and only 4-5 will turn up because they don’t care they will be like is there money or signing. Most people care about the money and if there is signing they will come in late to sign that’s why I prefer peer-peer.

*I: You have talked about money, do you think money can be a motivator?*

R: I doubt.

*I: Why?*

R: Like I have told you they will be money minded only for example to going to plan that we meet at 9 o’clock for the LCG and we shall have a facilitation so everyone will call their friend to attend because there is money, here their interest won’t be in the LCG but money so you will teach and if you give them this paper to fill at the end of the day you will be surprised that’s why I feel that those who are eager to learn will always learn even without money.

*I: What challenges have you encountered while using this tool?*

R: Very many challenges, here we have a challenge of limited beds so you’re going to admit your mother by the time you come for the next examination so this mother has no be and we can’t even listen to the fetal heart because the mother is just outside moving around you try to trace her and can’t find her so find the LCG is empty, you can’t fill in anything because the mother will come again when she wants to push.

Another challenge is since this is a teaching hospital you will make your own plan as a midwife because I have examined the mother and then there comes another person let’s say the 1^st^ resident doctor and they also examine and make their own plan and that brings a rope pulling/disagreement for example from your examination you would want to augment but from the doctor’s examination he wants to wait yet the mother is prolonging and after sometime that doctor tells you to wait for the 3^rd^ year to make a decision yet the mother’s life is in danger. So the problem is that you can’t make your own plan and implement it successfully.

*I: Which other challenges?*

R: I might end my shift when I have recorded part of the LCG and then there comes in someone who has knowledge gap so you come back to find that the other person didn’t fill in anything when the mother actually delivered and on the LCG nothing shows that the mother actually went even in the 2^nd^ stage but you find delivery notes so there is still knowledge gap amongst the people here.

*I: What do you say about the layout/design of the LCG?*

R: It is very challenging for the people who have eyesight issues because the words are very tinny so it’s hard for people with sight issues to see these things and plot them well. That’s the only problem otherwise the layout is okay because it has the key, the instructions and everything that is needed.

I: What would you change on this tool?

R: I wouldn’t remove anything and I don’t think I would even add anything.

*I: Based on your experience, are all these sections equally important or there those that are more important than the others?*

R: All these are important because I realized that’s why we have that change, at first I thought this supportive care is not needed after all this Africa and this is a developing country so I was wondering why I would even bother to ask about the pain relief but later I realized that its actually the most important because like companion psychologically if this mother is not psychologically okay trust me even the labor will prolong.

*I: Labor monitoring with the partograph would start at 4cm but with the LCG it starts at 5cm, what do you say about that change?*

R: I think the change is good because assuming someone has some tinny hands, they would plot 3cm for 4cm but here you can’t lie about 5cm so I think it is a good change if at all anyone has plotted 5cm wrongly at least it will be 4cm and the mother will surely be in active labor.

*I: What knowledge have you gained after using the LCG?*

R: I have learnt that anything about the mother can change anything about her labor progress for example I have told you if the companion is not there trust me things might change completely.

Another thing I have learnt that it is very important to monitor this mother in second stage before I wouldn’t care if I have filled my partograph and the mother is fully dilated but because there is a part that forces me to monitor this mother I have to monitor her and I have realized that it is very important because many mothers get challenges because they are not monitored in second stage.

I have also learnt that it is very important to involve the mother and her companion in labor progress.

*I: What changes have come with the introduction of this LCG?*

R: I haven’t seen any changes really and it looks that we haven’t fully focused on this LCG as a department but for the people that are committed to it, there are some changes for example like I told you mothers’ no longer get very prolonged labor because it will be detected early because decisions are made earlier.

*I: What do other people such as your work colleagues say when they see you using the LCG tool?*

R: Some feel the argue to learn using it when they see you using it, so far I have taught like 3 people some are still holding on to their negative attitude, some would want to learn but they are very lazy.

*I: Would you recommend that this LCG be rollout in all facilities in Uganda?*

R: I highly recommend it especially in our local facilities because here we always get obstructed labors so may be this would help those if they are taught very well they would make their plans and implement it immediately rather than leaving the mother to get prolonged because they also have the mentality that you have to take action after the mother has crossed to action line so if they learn that you can take action immediately trust me they would do better.

*I: On rolling out this LCG, what are some of those things that the government should bear in mind to achieve active utilization?*

R: They have to make sure that stationery is always available because we also have challenges with our government for example now we haven’t had admission forms for about a year so I am wondering if they get rolled out and then they are not supplied what will happen so they have to ensure availability of stationery. Then they also have to make sure that the people who are supposed to use them are equipped with enough knowledge. Then I don’t know how they are going to change the attitude of the service providers because attitude is a very big factor.

*I: What do you think about introducing this LCG in nursing training schools?*

R: That would work out like magic because students are always eager to learn and they are also forced to learn because they want to pass the exams so they must learn it so I think if it is introduced in schools it will work out very well. Thank you for that reminder.

*I: What is lacking in this facility to make labor monitoring better?*

R: Like I have told you we don’t have enough space, in labor suit we have 3 beds and they are always full, they are conducting deliveries from there. In admission we have 2 beds which are normally occupied by emergencies, then we have only 1 bed for examination and it’s also normally occupied, sometimes we use the only one bed meant for antenatal on postnatal mothers so it’s very hard to monitor mothers who are just outside loitering around I wish they expand.

Another thing I won’t say we don’t have enough human resource, we do have human resources but I don’t know whether its attitude and how they can change it.

Another thing is, for this thing to work out well the health service providers have to be motivated. They have to be cherished for what they are doing, because sometimes you spend the whole night in the labor suit monitoring mothers then you hear someone stating that us health workers don’t want to work so we should leave and you start wondering whether there will be a time when people will appreciate the work of health workers, we watch Tv and see people appreciating health workers that would give us encouragement to work even better but hearing trash about us!! I feel even our attitude would change if we are appreciated.

*I: How would you want to be appreciated?*

R: For example we have very many residents hear, if you hear them complaining that they don’t have money for forms how do you expect them to come and serve, they will just come and sleep. You hear midwifes demonstrating and how do you expect them to serve when their issues are not handled so they need to think about their personal wellbeing and then also they should always put in the CME’s they no longer do that, every time they say they don’t have money. Then also there should be some scholarships for people to learn more, some people are eager to learn but they have no resources.

*I: Is there any other thing you would like to tell me about our discussion?*

R: I want to appreciate everyone involved in this research and the introduction of the LCG because you are making sure that everything changes for the betterment of our mothers.

*I: We have finished, thank you so much for your time.*

**Participant ID:                                  CL010**

**I:                                                        WT**

**Transcriber:                                     KP**

*CL010 is a female health care work at Mbarara Regional referral hospital he thinks the labor care guide is easier to plot compared to the partograph and she actually feels that the midwives have been taking it up. She appreciates the fact that the labor care guide eliminates the prolonged daily latent phase because nose active labor is considered at 5cm rather than the 4cm that used to be considered while using the partograph. However, she cites a few challenges such as lack of training, shortage of resources such as stationery and negative attitudes towards the labor care guide tool. She suggests that if the tool were to be integrated in all health facilities then there should proper training that actually goes up to nursing training schools, provision of proper training and some incentives to improve the attitudes of the tool users and enhance its easy adoption.*

*I: Based on your personal experience what do you think are the recommendations or what do you think needs to be changed? That’s what our discussion is all about. So I would like you to start by telling me about your experience with the new labor care guide. How do you find it?*

R: First of all compared to the partograph we had the labor care guide is easy to plot because it has no those graphic things and another thing I appreciate on the labor care guide is, for the partograph we used to get prolonged daily latent phase because we used to consider 4cm at active labor and sometimes we used to take the 4cm even for the multiparous mothers so at least the labor care guide eliminates that when you start it at 5cm automatically you’re sure that the mother is in active labor.

Another good thing with the labor care guide it tells you the management because it has that part for the management you’re giving the mother at a certain stage although the other partograph had that part it was behind so it wasn’t directive and honestly people are taking up the labor care guide.

*I: Why do you think people are motivated to use the labor care guide?*

R: I haven’t asked them that but it’s an observation, I have observed that they are taking it up so I am likely to give a wrong answer on the why they are taking it up because I have never asked them but maybe they could be the same things I have told you. That’s what I am thinking.

*I: What challenges have you encountered in using this labor care guide?*

R: The challenge is that not all the midwives are trained so those who didn’t attend the first training start it but they don’t complete it and I think it’s because not all of them were taken for training to use it. Then another challenge there is a problem of paper work, you know with the shortage of resources sometimes we don’t have stationery so sometimes they want to use it but you find that there is not enough stationery. Those are challenges that I have observed so far, so if they can increase on the awareness and the knowledge. And also there is a challenge of the outside students because it seems the labor care guide is limited to within mbarara, it hasn’t reached to the outside training schools so the students who come for the practicum don’t have any knowledge of it so there is need to expand knowledge to the nursing training schools there we can do better.

*I: How does lacking of training amongst the staff or some midwifes affect the implantation of this tool?*

R: It affects in the way that as I have told you that those who start it don’t normally complete it. Then another issue is that you never know sometimes they say that an attitude is hard to change but when someone has knowledge it can go hand in hand with attitude so lack of training also affects attitude.

*I: For you, how did you come to know how to use the labor care guide?*

R: We were trained.

*I: Hearing about the labor care guide for the very first time! Where were you and how did you feel about it?*

R: I was told by the SHO for the first time and when I heard about it I also peaked interest to know the paper that was going to replace the partograph so the SHO is the one who took me through. Then after that we were called the grand training which I attended and we were taken through.

*I: How easy or difficult was it for you to use it?*

R: It wasn’t all that difficult but you know when something is knew you usually take some time to absorb it.

*I: How would you compare this labor care guide and the old partgraph in terms of monitoring the progress of labor and recording the outcome of labor?*

R: As I have earlier said the labor care guide removes the prolonged latent labors that we used to have with the partograph. And another thing the labor care guide has the alert signs/indicators so usually it brings out the problem quicker than the partograph where we used to straggle trying to interpret. For the labor care guide if you use those indicators it automatically tells you the problem.

*I: What do you say about the design and the layout of this labor care guide?*

R: The design is okay only that I have a little issue with the plotting of the irritation where we plot in between the boxes, I have been wondering whether it’s either before that time because you write the time in between that box so the plotting doesn’t exactly tell you at which minute did you do the plot but the rest of things are okay.

*I: That point is noted and I surely I think it’s going to be addressed.*

R: Then another issue is on the part of the second stage where to start putting the p’s where the mother starts pushing, that place is not direct for everyone to know that you’re supposed to put the p’ here, some put the p’ down, some put it up. So the place is not clear, I think it either needs to be bolded or generally there should be something showing that that’s where the p’ for the second stage is supposed to be put.

*I: What changes would you suggest to be made on this labor care guide?*

R: I feel the thing is okay. As longs people know it and understand it.

*I: In the sections of the labor care guide which parts are more helpful or more relevant in labor care monitoring compared to the others?*

R: Compared to the partograph or compared to the other components of the labor care guide!

*I: Compared to the other components of the labor care guide.*

R: Each component has a different purpose from the other so they are all important. I won’t tell you that it’s the part of the fetal monitoring that is more important and I leave out the progress. Each component has what it monitors or its importance and each component is serving its part.

*I: How did you feel when you were told that the new labor care guide was going to replace the old partograph?*

R: As I have told you when something new comes in the place you first see it as a challenge but after getting enough knowledge you clear your minds. So at first we saw it as something that was more complex but after getting the training we realized that it was a simple thing.

*I: So the training helped you a lot adopting to this?*

R: Yes. It’s all about training.

*I: Is there any time when you didn’t use this labor care guide during your time at the hospital?*

R: As an individual or as a ward?!

*I: You as an individual.*

R: It’s not like I monitor mothers every day because I do more of administrative work so I can take some good days without monitoring mothers but whenever I have a mother I use it. So I won’t say that I use it on a daily basis yet sometimes I do administrative work. Now if ask me on behalf of the rest of the ward I can give you feedback on that.

*I: Okay, on behalf of the ward is there time when they have not used this labor care guide?*

R: Yes, there are some cases where a mothers comes as an emergency cesarean section so the mother is taken without starting her on the labor care guide. Then also as I have told you it depends on the midwife who is on duty, if the one on duty was not trained on how to use the labor care guide, you will find it either incomplete or they will not start it.

*I: What do you as a clinician need to make the monitoring of labor better at mbarara regional referral Hospital?*

R: The availability of the tool, if the tool is there in time then it can help in monitoring and another is the training as I have told you, they need to bring everybody on board.

*I: What do you think about adopting and integrating this new labor care guide across all hospitals in Uganda?*

R: They are even taking long to adopt it because I have told you that we are facing the challenge of the outsiders, when they come they don’t know it and when we try to take them through sometimes they think that it’s the thing for mbarara so I feel they are taking long to adopt it. They should adopt it immediately and when they adopting it they shouldn’t forget the training nursing schools as I have told you earlier because that’s where we get midwifes who come for trainings and they are without any knowledge thus teachers should also be trained.

*I: Any other thing you would like to say apart from training?*

R: I think it’s only about training, I have talked about training and availability of the tool.

*I: As an administrator what would you advise people who want to integrate this tool, I mean what are those things that should be in their minds as they are trying to integrate it in all hospital facilities?*

R: One thing is about the attitude. As they are trying to integrate it, they should think on how best they can change the attitudes of people so that they can love the tool and use it. If they don’t think about it you can never know what happens. Then secondary it’s the training.

*I: What are some of those things do you think can be done to change the attitudes of these individuals?*

R: One is training because I believe that if someone has the knowledge the attitude can change a bit because some pretend that they don’t know. Then another thing is supervision because if you implement something and you don’t supervise it to know whether and why people like it or they don’t like it then you won’t get the ways of improving people’s attitudes. Through supervision and mentorship, I think they can improve on the attitudes.

*I: What challenges do you think this labor care guide might face if we are to implement it as it is especially in the Ugandan hospitals?*

R: Availability of the tool/papers because we have challenges with the stationery so if you implement it without looking for solutions, you might implement it and we fail to get the tool.

*I: Is there any other thing you would like to tell me about the discussion that we have had?*

R: I think I have exhausted everything according to your questions, maybe I can emphasize the issue of attitude it needs an immediate solution because I feel it can hinder the implementation. I mean if people fail to like something whole heartedly it can be challenging. Thus there a need for an immediate solution otherwise we can get all the resources to use the tool but people fail to like it.

*I: In addition to training, do you think motivations in terms of incentives can change their attitude?!*

R: Yes, I won’t shy away from saying this. I think incentives can do something, an incentive might not necessarily be money but something that can be put up to recognize people who are doing better, it can encourage those ones who are not doing the thing and it can change the attitude.

*I: How about the finances!!*

R: The finances wouldn’t have a problem but my concern is on the sustainability. For how long will you give that money and how much will you account for the work? So that’s the problem with money, if you give me today and tomorrow you don’t give me then the next day I won’t use it because you didn’t give me money. That’s why I am saying that we can look for other ways of appreciating their work and recognizing good performers so that they weak ones can also learn something. Money can be turned into something that can motivate them.

*I: Is there something you would like to add on?*

R: No that’s all.

*I: Thank you so much for your time.*

**Participant ID: MOH001**

**I: WT**

**Transcriber: KP**

MOH001 is a female district health officer who likes the parameter measurements of the labor care guide because it separates contractions per 10 minutes, she also appreciates the section of making decisions and also appreciates the sections of initials as it provides ownership to those who would have plotted the labor care guide but she feels it isn’t good to leave out the action and alert lines since they have been good at guiding midwives while using the old partograph, also she feels the current printout of the labor care guide tool is too congested so it would need some revising. If the labor care guide tool were to be integrated in all health facilities, she feels there would be a need for comprehensive training of all midwives, supervisions and monitoring as well as incentivizing its use to motivate its users and make it a success.

I: Thank you so much for accepting to be part of this, there is a new labor care guide I don’t know if you have heard about it or you have come across it?!

R: I have heard about it but I haven’t used it.

I: This is like just like a pilot study intended to pilot this tool in public hospitals so that it replaces the partograph so this labor care guide has 7 main components that involves the patient’s identification just like the other partograph. It captures details such as the names, age, and the weeks of gestation and then it has the risk factors then this adds the syphilis and the hepatitis B status. Then it also has the stages of alerts the 1st and the 2nd stage. Then it has the supportive care section that talks about the companion, the pain relief, the oral fluid and also the posture then it has a section about the baby this talks about the baseline fetal heart deceleration, the ammonitic fluid, the position put and molding. Then it goes to the woman and discusses the pulse, the historic BP, the temperature, and the urine. Then it also goes to the labor progress, it tracks the contractions especially per 10 minutes and also the duration of the contractions and then also the cervix, then it goes to medication this talks about the iv fluids and the medication given to the woman in labour. Then it talks about the shared decision making regarding the assessment and the plan. And then the very last part has the initials of the person filling the labor care guide, then it has the outcome of the labor including the date and time of delivery and more of such details.

So in this study we would like to know how health care workers feel about this labor care guide. So even though you have not yet used it based on your knowledge, what do you have to say about this labor care guide?

R: What I like about it is the parameters measurement I can see they are trying to separate the contractions per 10 minutes as a separate row from that one of the duration of the contractions because in the old partograph that would be combined, people would just be guessing but here if it means people documenting the time then they will have to be serious and put the time which will make a better a assessment than someone just shading the whole box without measuring.

Then another thing I like about it is this section of making a decision so after measuring you make a decision although the space for writing is very small I don’t know how many people can write legible things in that box. Also the issue of initials is very good because sometimes people plot and there is no ownership unless you see the behind part of the labor outcome but usually there is no ownership of that first graph but if it is plotted by two different people you will see where someone started and stopped and where another person picked from.

But I wonder why they left out the action line and the alert line that quite challenging because these have been good in guiding midwives to see when they should start getting worried, they would see that line on the partograph and then they start being uncomfortable but here someone can plot continuously without thinking of what to do next. For our lower cadre midwives, in case there is overload where someone is dealing with many mothers and doesn’t have time to critically look at each, some mothers might exceed the time because midwives are already used to that line and it has been helpful.

I: Is there any other thing you would like to say about this?

R: Again the way they prepared this section for fetal heart monitoring, you see they just put numbers for example you can record a 100 and so on, in the old partograph you would draw like a graph and when someone looks at it from a distance they would quickly know that there is a problem either the baby’s heart is continuously going up meaning there is a problem or it is declining but with numbers you need to think through to know whether your progressing well or you’re not progressing so I don’t find this kind of plotting nice for someone who might be busy, they might not quickly tell that the baby has a problem. Now if I write 120, after 20 minutes someone writes 110 then another one comes writes 160!! If it’s a graph you will just look at the range of this child but with numbers you have to sit and think so it doesn’t come out so nice. I would prefer the graph for fetal heart rate monitoring.

I: So, do you adding a graph to this labor care guide would make it better?

R: If they put a graph like it was on the partograph, put boxes with numbers which I think start from 100 onwards and I think there is a level where you exceed and know that your no longer in a good place, there is a place where your plotting and you know that you’re comfortable as long as there graph is in between there you know you’re okay but this thing of just writing numbers for monitoring is not okay.

I: In your opinion, how best do you think that approach of writing numbers can be improved?

R: It can be improved if they put something like a reminder in brackets they put the normal range that is expected so that if something is getting out of the normal range the midwife or the doctor writes in red color so that whoever comes knows that there were abnormal ranges detected it may not exactly show the trend but it can help if someone is getting out of the range and you write the numbers in red it can be quick for someone who is taking over.

I: Do you think with this labor care guide all the parameters adequately required to monitor the mothers in labor are provided?

R: I don’t see the urine output.

I: The urine is under is under the woman section.

R: I have seen but if urine is under woman, it is when they are testing to see whether the mother has proteins but it doesn’t show the volume for example whether the woman is urinating because you can test without measuring. Maybe if there was another box of the output under that box, it’s very important especially for mothers who have complications.

I: Is there any other component in addition to that one of urine?

R: I think those are my quick ones since I have not yet used it personally, I am just comparing it with the partograph.

I: What do you say about the layout and design?

R: The layout is good I think it’s also on one page, the challenge is I don’t know if it can be printed out properly because the one I have seen is so squeezed so that the boxes are a bit big and people are able to write comfortably.

I: The old partograph would start monitoring labor at 4cm but this one talks about 5cm, what do you say about that change?

R: I think it’s not a good thing because already we have mother who can be at the facility for 2 days in latent stage before reaching 4cm so if wait to plot the partograph at 5cm sometimes it might be late because if someone started plotting at 4cm it would help them to monitor and see may be within 4 hours they would expect that mother to be at 8cm and if after those 4 hours they haven’t reached 8cm then you know that may be they are having a problem. Now if you delay and want to start plotting at 5cm it means you will cause a delay somewhere for the intervention, that’s what I think but may be if we got more training we would know why they opted for 5cm. in our limited resource setting you have to plan to move the mother early because we don’t have all the services needed, considering the time they have to be travelling then they have to wait even the other side so for I think delaying to start plotting isn’t good.

I: What changes do you think will be brought by this new labor care guide tool especially to help facilities in regards to monitoring of labor?

R: Like this section I have already talked about of shared decision making after you have recorded whatever you have recorded then you have a plan you assess and make a plot so I think that will bring a great impact on how health care workers are using the labor care guide. But some people just wait until the mother has delivered then they start plotting after the mother has been delivered this may encourage them to start writing everything early in real time, so it will make them responsible.

I: Based on your knowledge as a senior health officer at the district, if this labor care guide was to be rolled out in all health facilities may be the government wants to adopt it. What are some of those facilitating conditions do you think should be in place, or you feel can enhance the utilization of this guide?

R: We have always had a challenge supporting midwives and other health care workers to use a partograph, it’s really not something they would love to do so you have to keep talking about it. They had improved just of recent because of the program which was based on paying based on the output meaning if you not done it then you don’t get paid, the facility scores less then you don’t get money for incentives as health workers so that was the time I saw that everyone was very serious with it so I think recognizing and praising people who using it very well can motivate people. You find there people who plot things well whenever they are on duty and there are those who just write their names so I think also using it as a performance measure.

I: You have talked about the idea of being paid for the output, do you think that can also help?

R: It (RBF funds for incentives) had helped because we would assess how many deliveries you had and give a score for that then how many were monitored using a partograph, we would critically look at the partograph just in case there was anything missing even if you would miss one thing you would lose a mark. That used to help health workers to be committed to making sure that everything is documented so it had helped because it was incentivized so they would put a focal person to make sure that everyone is filling it the right way. They were doing it for money but I think as a district if we had funds to help us visit all those facilities at least once a month and closely monitor them because they used to do it knowing that we are coming to monitor them so I realized that even though there is no money but they health workers know that the DHO will come to monitor them, they will do it well so I think there is need for intensive appraisal and also close monitoring.

I: In addition to supervision and monitoring, what are some of those things can the ministry of health can ably do to make sure that the utilization of the labor care guide is successful?

R: Training all midwives to make sure that all midwives get firsthand information. You see in most cases they train one midwife to go and train the other 3-4 midwives so sometimes the other 3-4 feel like this one who went and maybe got the training allowances should do the work, even if she tries to train them, they won’t be attentive. If there was a chance where by the do phased trainings and make sure that every midwife has attended and learnt how to use it then it would help, this eliminates the excuses of some of them saying that they don’t know. Much as we blame them some of the midwives being trained now or even before have not had a good chance to use during school moreover this one is new, so sometimes when you come and take them through quickly for one hour some people will not appreciate it well but if they are given time and scenarios during classroom training so that people feel knowledgeable because sometimes people something because they don’t feel knowledgeable or skilled so if they feel confident then it will push them to use it. So I would request for trainings and on job mentorship and also making sure that the tools themselves are available and user friendly not like this one I see here that I feel one has to use a microscope to read it.

I: Apart from providing those printouts, are there other logistics do you think can be provided to health care workers to use this tool?

R: I think that one is done well it will do, if the tools are available then it will be fine.

I: What are the likely challenges that the utilization of this new labor care guide might face even when the government has decided to roll it out?

R: I think now people are not using it because they feel it has not be officially released by the government so if it is officially released it will be used but I think if you ask the others they will give you the other comments I gave at first especially on the changes of plotting and the alert lines because they have been helpful, some old midwives would want to stick with the other one because it helps them to stick with the one because it has been helping them to quickly see the problem. In this I think when they train they will tell people how they can quickly identify a problem. The side of the child you can tell but this side of the mother is not so clear for me.

I: Do you feel those old midwives might resist it?

R: Yes, they might resist this just because of some of those things which are lacking may be if the training gives them an alternative of how to identify those issues but if the training is not comprehensive then it won’t be good.

I: Based on your assessment of health facilities in Mbarara and their managerial and administrative needs, do you think that health facilities are ready for the roll out and integration of this labor care guide?

R: If the training is done they will be ready because the other things are what they have been doing so they are ready.

I: Is there any other thing to talk about based on our discussion?

R: May be the 1st section has some little space like when they put date and time, I think the partograph has time of admission and then time when labor starts so I think they need to specify those times also.

I: That’s noted, is there any other thing?

R: No that’s all.

I: Thank you very much we have finished the interview

**Participant ID:                                   MOH002**

**I:                                                        WT**

**Transcriber:                                     KP**

*MOH002 is male Ministry of health representative who thinks that the integration of the labor care guide can genuinely be guided by the research findings thus he appreciates the researchers who are taking the initiative to find out. And he feels if at all findings prove that the labor care guide is a reliable tool then the government is ready to roll it out, it’s just a matter of training and mentoring front line health care workers to have them equipped with comprehensive knowledge and skills. To him training and mentoring the already deployed front line health care workers about the labor care guide tool is more sustainable and main streaming.*

**I: Like I have said the labor care guide is made of seven main parts it’s more of a partograph but this is a bit detailed. It has sections about the social demographic details of a patient but this time it captures syphilis and then the hepatitis B, then it has sections for supportive care in response for the companion or the next of kin, then there is a part for the baby and then a part for the mother, there is also the progress of labor and then people can put their initials to show who has monitored and if the identify a problem they can circle or highlight.**

**This is a tool that we are trying to pilot test so based on your ideas with the old partograph, what do you think about such an intervention?**

R: I think it’s good and I think from the ministry of health we want to see what the frontline health workers see I can’t now answer questions that are likely to influence decision making because we have to know what the front liners think, we want to know why we are still struggling with the intrapartum is it the tool, is it the attitude!! Those are the questions that need to be asked. Is it that the partograph is not comprehensive enough to support intrapartum I think Dr. Mugyenyi’s research findings are supposed to help us in the policy perspective. Truly we have been using the partograph for many years but we need to change because the evidence we got from the study is telling us that we should change. That’s what we should think about. The tool is very comprehensive so we want the front liners’ to tell us its usability, it’s more detailed than the partograph, is it implementable! Are we just making it work! For them to use a tool more detailed than the partograph so we are waiting to see, the people who proposed it were saying that lets pilot it here and see what the feedback will be.

**I: On the policy level and also knowing the nature of our health facilities, what are some of those factors that you feel can facilitate the implantation and utilization of this new labor care guide?**

R: The very first thing are the findings of the study if really the findings are very solid that we need to switch from the partograph to the labor care guide, the rest is easy. The other factors would be if we had the resources available to train people and scale up, if evidence tells us that we need to switch we shall switch. There are no complex factors, we just need evidence to inform our decision as we moving from long used partograph to the recommended new LCG. This is going to give us findings that are very critical.

**I: How is the readiness especially of the ministry of health?**

R; They are ready the government is ready to receive the information but I think right now the ministry of health is taking a more evidence-based approach, that’s why we are partnering with academia, we want local evidence to inform policy, so there is credible willingness.

**I: Going to the role of supervision and monitoring from the ministry of health because as we introduce new interventions in clinical practice, there are those factors for example resistance to change. Yes the ministry is ready but it is one thing to put up an intervention and it’s another thing for practice to actually happen. So, what’s your opinion regarding ensuring practical usability especially when this tool?**

R: It’s during policy guidance, if at the center you agree that you have to practice this then the rest is easy. But before give the guidance we need the evidence because we don’t want people going around singing songs so we want some evidence that can enhance our argument as we engage hospital directors and frontline health workers as well. I know some studies have been carried out regarding the partograph and the labor care guide but we hope Dr. Mugyenyi’s study will be very presentable and help to build a very solid case. Of course change takes time and transition is a process but as soon as we have this then the rest is okay, there won’t be a problem.

**I: In the responses of the frontline health care workers some challenges emerge for example knowing that the overburdened health sector where by there is only one midwife on the labor ward with about 10 mothers that are all in labor. Sometimes they highlight that there is a gap especially that the labor care guide might require more staff, don’t you think implementing this labor care guide when the staff is not adequate would have effect on its actual utilization. You find that the tool is there but the actual real time monitoring is not done because of the nature of it?**

R: Is that what the study is telling us?!

**I: From the preliminary because I haven’t analyzed the findings.**

R: So, we shall need those results.

**I: This comes in form of a question especially concerning the ministry of health’s readiness because as we are asking, we also want to know the implementers’ side for example what is in place to ensure successful utilization or what are the, or what are the perceptions or what are those factors from the implementers’ point of view that they feel that if this tool is to be successfully implemented such factors have to be in place?**

R: Of course we have to review structure, the need to address the human resource gap, we have deliveries starting with health center III’s some of these things take a bit of time but just like a partograph has been in use a midwife or an obstetrician monitoring a mother you have to record your findings and that doesn’t necessitate a second person to look through your findings so from the ministry of health’s perspective I think they are supposed to address some of those gaps and we wouldn’t ignore the feedback that front liners tell us because it would influence the uptake of the innovation but now that we don’t have that information yet we can’t react. We are waiting to see in terms of what proportion of the health workers you would have interacted with are giving us what kind of information so the study is an opportunity to give us feedback.

**I: Is there like an incentivization from the ministry of health that is in place or that is anticipated to be in place to help this especially when it is rolled out?**

R: We are not incentivizing anyone, why would we!

**I: I am wondering why you think it’s not important!**

R: Because it’s not sustainable also by the time someone is going to use something it means there is a motive as to why it is being introduced as health care workers would want to be told that the tool, you’re going to use is something that is going to make your work easier. If you have been getting certain levels of mortality we expect them to go down, so we expect such a justification which will be part of our dissemination packages that we are moving from a partograph to a labor care guide for these reasons and not just throwing it to people’s faces, we have to accompany it with some reasons and those reasons must come from the study so we are not giving anyone incentives but we are telling them that based on the findings we are advised to move from the partograph to the labor care guide.

**I: Are there plans that are in place to facilitate the migration from the partograph to this labor care guide in terms of logistics such as papers?**

R: Right now we don’t have a plan but also we have stationed booklets which are for partographs right now for transition it means that we are switching and these booklets are printed so we switch, we use our available mechanisms, we utilize the available resource and replace the partograph, we print the iucs because to integrate means using the available mechanism it’s not running a separate thing so we shall have to use the available means to ensure that the labor care guide is available for use. In some areas some partners come in and then they bring some print outs but we need something sustainable so mainstreaming is the best thing.

**I: What are those things that you expect to get from health care facilities when they are utilizing this labor care guide?**

R: Improved intrapartum that’s what we want, I mean the reason why we would be taking up such initiatives is because we need reduced numbers of fresh still births’, reduced numbers of rapture, maternal mortality resulting from poor labor monitoring. There is nothing much we are looking for, this is supposed give better labor monitoring.

**I: Why has there been a delay in the ministry of health to adopt this labor care guide?**

R: Because we need solid evidence which Dr. Mugyenyi has now started, without it available we cannot make a move, that’s why this is being done as long as it is ready then we shall start staging some meetings to justify why the transition must happen.

**I: In the future do you feel like establishing focal people that are like champions in the ministry and also health facilities, do you think they can facilitate the smooth implementation of this labor care guide?**

R: It depends on resources, if we get a funding from partners, if we get a partner and he tells us that they are going to facilitate champions as part of our dissemination strategy that’s the best thing we can have but I think instead of saying that they are labor care champions we would want people to help us doing intrapartum monitoring so we want to package the deliverables of such individuals in such a way. Otherwise it means that we shall keep on deploying a number of individuals for specific tools which is not sustainable. But one thing that I am sure of is that we need to invest in the labour suites, we want to see how much can be invested in the labour suite because that’s where much of the trouble is coming from, so if it is identifying individuals that are intrapartum care champions that would be good but also we looking at these ,midwives who are already deployed and are within the structure, they have been using the partograph before but now instead of using the partograph they are going to be using the labor care guide so I think our priority would be to ensure that these same midwives have the knowledge and the skills necessary to use the labor care guide. Now that’s sustainable and mainstreaming as well as integration, for us that would be the easiest, cheapest and quickest route in terms of sustainable implementation.

Yes we have always wanted champions but how many champions are we going to get! Are we going to replace midwives with champions, if its dissemination and we want trainers then that is a separate conversation because you are able to settle out and know that you’re going to train this region in this period of time, you expect that the end of the training all the front line health workers who see mothers in labor should be competent in using a labor care guide and you expect the system to continue. And that’s the route we would always want to take, the one that’s sustainable responsive and resilient, once you strengthen the structures and system.

**I: We have talked about the need to invest in the labor suits because that’s where the problem comes from, I would like to know more information about that?**

R: We want to put a little more focus on the labour suite, monitoring labour is critical. MPDSR reports indicate significant mortality and morbidity around the time of birth

**I: Based on the labor monitoring policy and also your medical practice, do you feel the labor care guide has all the components and the parameters for monitoring labor or there is something that you would recommend that’s supposed to make it better.**

R: I think it’s comprehensive enough as it is because it is more detailed and you can hear more from the front liners about their thoughts in terms of usability.

**I: Do you have any other thing that you would like to share with me regarding this interview?**

R: Thank you, Dr. Mugyenyi and the entire research team for taking this challenge its very relevant because research is key to transformation so I think this is a very important study so we greatly appreciate the time you’re putting in and the findings that come out will be important because we are still struggling with high maternal mortality prenatal mortality is still very high and the and the biggest portion is resulting from the labor suit, so if there is anything that is going to improve labor monitoring then it is welcome.

*I: Thank you so much.*
