# Supplementary material for: Customization and acceptability of the WHO labor care guide to improve labor monitoring among health workers in Uganda. An iterative development, mixed method study
Source: PLOS Glob Public Health. 2024 May 13;4(5):e0002780. doi: 10.1371/journal.pgph.0002780 (PMC11090317; doi:10.1371/journal.pgph.0002780)
Supplement: Attachment — Submitted filename: Response to reviewers.docx [file pgph.0002780.s002.docx]

**Dear Reviewers and Editor**

Once again, I thank you for considering my work for consideration for publication at the prestigious Plos Global Public Health. On behalf of all the authors, I am excited and happy to provide detailed response to review comments as guided. The comments were sincerely very helpful and facilitated a comprehensive review of the entire manuscript to focus it to read better. We have endeavoured to provide a detailed point by point response in the table below.

I thank you for your technical guidance.

**Dr. Mugyenyi R Godfrey**

Corresponding author

**Please find the table below.**

| **Reviewer #1:** General observation and concern | Thank you for your constructive review comments. Below I present point-by-point responses to each raised concern |
| --- | --- |
| The study purports to make over 50 modifications to a WHO document. A good justification for such a major modification to a WHO document is needed. The aauthor’s should justify the value this would make considering that such documents from WHO are done which is usually evidence based. They should indicate also if there is need for the WHO to overwhelm such a document | This has been toned down. Important modifications were made on the section for identifying the mother and labour monitoring sections of the WHO LCG. Other modifications came as additional parameters to make the modified WHO LCG a stand-alone tool for managing labour in low resource settings. I agree that these modifications and refinements made on the WHO LCG should not create alarm but rather improve user centeredness for long term use in labour monitoring |
| The paper also needs a lot of focus. | The paper has been massively revised to focus it appropriately |
| **Background** |  |
| This need to be beefed up to include critical literature such as: Adoption of the WHO labor care guide in other settings. | This has been done in all the sections. More papers were read and used to beef up the literature on adoption. I however note that there are no many such studies reporting adoption and use of the novel tool |
| How much customization has been done in other settings and what has been the outcomes of such customization if any. | There are no reported customization studies done on the LCG so far. This gives our study strength |
| Studies to evaluate such modified LCGs through RCTs or pilot should be cited. | No such studies have been reported in the literature.  Joshua Vogel et al 2024 have reported an on-going study to evaluate the effect of the LCG in reduction of C-Section rates in 4 hospitals in India. This has been cited ref number 16  Pandey et all 2022 in their open-label randomized controlled trial on 280 low-risk antenatal women admitted for delivery at a busy tertiary care institute in North India showed significant reduction in Caesarean delivery rates among women monitored using a WHO LCG (1.5%) compared to control group. None of such studies customized the LCG (ref number 17) |
| The gaps that informed the current study is are also missing | This has been fixed Line 106 – 120  “Identifying and scaling up such context-specific interventions have great potential to improve quality of healthcare and outcomes in pregnancy, childbirth and immediate postnatal period [13, 19-21]. The Labour Care Guide manual has been developed by WHO to help skilled health personnel to successfully use the tool. However, the tool has not been adapted and adopted to be used as a decision-making tool in any low- and middle-income country maternity care settings, with correspondingly high rates of prolonged/obstructed labor, unnecessary interventions, maternal and perinatal mortality and morbidity. Whereas the barriers and challenges of using partographs are widely documented [22], there is little documented effect of the new LCG, and the entire implementation strategy has not been evaluated in low resource settings. There is also an urgent need for not only adoption studies, but also refining and testing an effective implementation strategy that not only addresses the current contextual barriers to labour monitoring, but also fosters sustained uptake and use of the new LCG as a recommended standard for providing and improving routine intrapartum care globally. In this study, we attempt to customize, refine and test the new WHO labour care guide among health care provider monitoring labour in South Western Uganda, as we explore a suitable/appropriate implementation strategy for the new tool in a Ugandan context.” |
| Study design: This section needs focus and quite a bit of detail. The design itself is not stated anywhere in the paper although it looks like a mixed method design. What is stated as a design is a rumbling narrative which sometimes looks like literature review and may not be replicable. | This has been clearly stated throughout the paper as mixed methods study. |
| The tool mentioned after deployment of prototype 2 should include how long it was tool used, by whom and to deliver how many women and how many facilities were involved. | This has been revised in all sections of the paper |
| A critical component of the LCG is labor and delivery experience of women? How this was assessed is not indicated. | In this study, we never reported the experience of women. We have mentioned this as a limitation LINES 518 -523  “Our study also recognises some limitations. We did not evaluate the feasibility, rate of completeness, incremental cost, sustainability and effectiveness of this new prototype to improve maternal-foetal clinical and labour outcomes. **For this particular study, we assessed experiences of LCG amongst HCPs, but did not assess the labour and delivery experiences of women. Evaluation of our final prototype is ongoing to document effectiveness and its diagnostic validity compared to the partograph”** |
| In all the narrative in study design the sampling and proportionate allocation of HCWs at different data collection points is lacking considering that they have different characteristics. | This has been clearly revised throughout the whole paper to include allocation of different HCPs in different sections describing different data collection points |
| The same is lacking at the stakeholder level considering that not all persons attending the 2 mentioned conferences could be interviewed. | This has been revised. The section describing the expert opinions at the conference were removed from text, since it was not possible to describe persons attending the conference |
| Also, a statement “The final feedback was obtained following a comprehensive presentation” is given but it is not clear who gave this final feedback after the use of prototype 3 and how the participants were sampled among the users from MRRH. | This statement has been removed from text |
| The tools that we used to collect data to assess critical components at each step needs to be mentioned | These have been stated in each section for example  …Expert panels used self-administered structured questionnaire line 214 with interviews and FGDs utilizing interview guides |
| Data collection is just mentioned in passing in a line. This needs proper elaboration for duplicity. | This has been better described through the paper |
| The study setting is too long and should be summarized | This has been summarized, but maintained critical sections necessary to describe the study population to facilitate understanding of sampling of HCPs and stakeholders interviewed |
| The show of “prototype development and pilot testing” needs to be clear and is it should be clear how pilot testing was done and what happened next. | This has been done |
| It should be indicated how many stakeholders were interviewed and how were they allocated to each cadre who then used the LCG. It should also be clear which were the 4 facilities that this was done and how were they chosen. | This has been clarified |
| In the HCP exit Interviews and FGDs word used here such as “At least” , “of different cadres” and 10 HCPs of “all cadres” are too general and unspecific. It is also good to indicate the timeline. | These have been revised to read  .. Two FGDs were done to include 10 HCPs each of all cadres that utilized prototype 2 to monitor labour for at least two months in the three public facilities discussed above. These FGDs were conducted to explore experiences, gaps and obtain feedback on the modified prototype 2. This input/feedback was synthesized, and ranked to refine components of a subsequent prototype 3. |
| **Results** |  |
| This needs a lot of focus. The current result narrative looks like discussion with even a citation “31”. Most of the results in some narrative lacks data to support and most are supported by only 1 quote from the qualitative aspect. | The results section has been revised as suggested, we have also added more quotes on most results as you suggested |
| Before table 2 it would be good to have a table that shows the output of what lead to the changes that were made and shown in table 2. | These have been provided but in narrative and not in table format |
| In the segments “Customizing LCG by adding key socio-demographic data compatible with existing programs” the narrative cannot be supported by only 1 quote. There should be some results shown in a graphical or tabular form. | More quotes have been added to beef up the results. This has been done in most of the results |
| The many modifications done in “Modification of key fields to suit local context” should really be justified as a WHO document has gone though a lot of iterations and uses best science. Also, what appears here is actually discussion and not results. | This section has been revised |
| The issue of discussion happens in most other sections. The input that led to all the changes mentioned here should be very clear. | We have revised the discussion to discuss the results, thus focusing entirely on the results |
| The same lack of data and narrative looking more of discussion is seen in “Inclusion of key clinical notes and labor outcome data” A table 4 is also mentioned but is not seen in the document. | This was been revised as recommended. Table 4 was quoted in error and has been removed from text |
| Table 3 is poorly done with many cells that are having zero frequencies (0%). A bar graph would be a better way of presenting such results. Also, the narrative is more of a discussion than results presentation. | We have not been able to use a bar graph to replace table 3 because the variable reported are too many which would not make a smart bar graph |
| **Discussion and conclusion:** |  |
| Since the results are not clearly presented, the discussion is speaking to results that cannot be verified as in paragraph 2&3. | These have been rectified |
| Comparisons of results should be with similar studies showing similar assessed variables unlike what study cited as 34 is where authors are discussion time of filling the LCG and cites a study on outcomes. | This has been rectified |
| Paragraph 3 is mainly speaking of data that is not presented. It would have been good to have utilization data. | This has been rectified |
| What is in paragraph 4 is more of methodology and should be taken to the relevant section. | This has been done |
| It is not clear how the variables that are mentioned in the last sentence were evaluated and what the results were. | This has been rectified |
| Paragraph 5 has also a lot of repetitions and methods. With the data presented the conclusions that the modified LCG is “simple, appropriate, easy-to-use, promote HCP-labor companion-patient interaction, responsibility, accountability, team work and confidence among HCPs” are not supported | This has been rectified |
